# Group-I lead oxide X_2_PbO_3_ (X = Li, Na, K, Rb, and Cs) glass-like materials for energy applications: a hybrid-DFT study

**DOI:** 10.1039/d5ra07886e

**Published:** 2025-12-18

**Authors:** R. Zosiamliana, Lalhriat Zuala, Lalrinthara Pachuau, Lalmuanpuia Vanchhawng, S. Gurung, A. Laref, Shalika R. Bhandari, D. P. Rai

**Affiliations:** a Department of Physics, Mizoram University Aizawl-796004 India dibyaprakashrai@gmail.com; b Physical Sciences Research Center (PSRC), Department of Physics, Pachhunga University College Aizawl-796001 India; c Department of Physics and Astronomy, College of Science, King Saud University Riyadh 11451 Saudi Arabia; d Department of Material Science and Nanotechnology, Nepal Academy of Science and Technology (NAST) Khumaltar 44700 Lalitpur Nepal

## Abstract

Pb-based compounds have garnered considerable theoretical and experimental attention due to their promising potential in energy-related applications. In this study, we explore the glass-like alkali metal lead oxides X_2_PbO_3_ (X = Li, Na, K, Rb, Cs) and assess their suitability for piezoelectric and thermoelectric applications. First-principles calculations were performed using hybrid density functional theory (DFT), incorporating B3LYP, HSE06, and PBE0 functionals. Among these, PBE0 is identified as the most accurate, yielding lattice parameters in close agreement with experimental data. Structural stability was confirmed through the evaluation of thermal, mechanical, and formation energies. For the non-centrosymmetric orthorhombic phase *Cmc*2_1_-X_2_PbO_3_ (X = K, Rb, Cs), piezoelectric constants were computed *via* both the numerical Berry phase (BP) method and the analytical Coupled Perturbed Hartree–Fock/Kohn–Sham (CPHF/KS) formalism. Notably, Cs_2_PbO_3_ exhibited a piezoelectric coefficient of *e*_33_ = 0.60 C m^−2^ (CPHF/KS), while K_2_PbO_3_ showed *e*_32_ = −0.51 C m^−2^ (BP). Thermoelectric properties were investigated using the semiclassical Boltzmann transport theory within the rigid band approximation. The calculated thermoelectric performance reveals promising figures of merit (*ZT*), ranging from 0.3 to 0.63, suggesting these materials are applicable as future thermoelectric materials.

## Abstract 2

Glass-like alkali metal lead oxides X_2_PbO_3_ (X = Li, Na, K, Rb, Cs) are explored and their suitability for piezoelectric and thermoelectric applications assessed.

## Graphical abstract

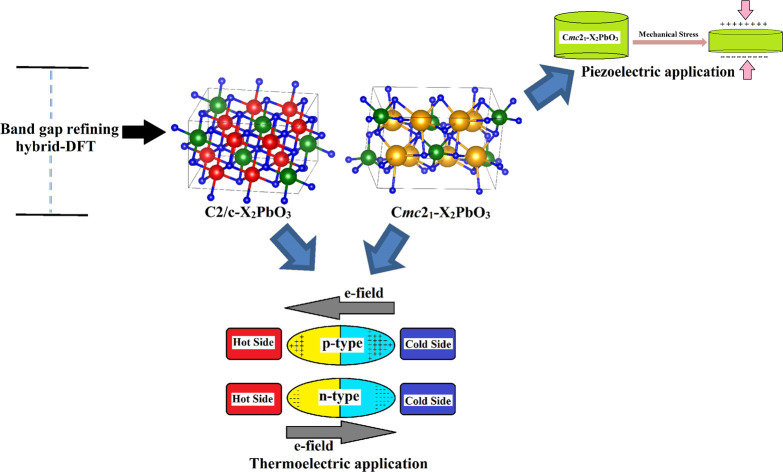

## 1 Introduction

Energy generation and storage is crucial to meet the current global energy and environmental crises. The energy extracted from fossil fuels is extensively utilized for transportation, many industries and many other purposes, despite its known harmful effect.^1^ Thermoelectricity holds promise as a suitable and sustainable alternative to greenhouse gases emitting fuels if efficient thermoelectric (TE) devices of high heat energy-to-electric voltage conversion efficiency can be discovered.^2–6^ First-principles calculations with the incorporation of Boltzmann transport equation *via* the BoltzTraP code^7^ can help in predicting the candidates for the preparation of TE devices. Glass-like compounds have become fascinating materials, both experimentally and theoretically, due to the complexity of their structures which in turn exhibit low lattice thermal conductivity, tunable electronic properties, and flexibility in fabrication.^8–11^ Although silicate (SiO_2_) glasses are widely abundant and commonly used materials, they have significant limitations such as brittleness, high resistance and phase transition at higher temperatures, *etc.* Therefore, it is pivotal for researchers to find proper replacements for silicate glasses.^12^ In this novel work, we propose alkali metal oxide Pb-based glass-like materials X_2_PbO_3_ (X = Li, Na, K, Rb, Cs) as potential replacements for SiO_2_-glasses such as: Li_2_SiO_3_, Na_2_SiO_3_, *etc.*, and conduct a thorough investigation on these compounds using density functional theory (DFT), particularly for their TE applications. To the best of our knowledge, among the suggested alkali metal oxides, K_2_PbO_3_ was the first compound to be synthesized back in 1964 by Hoppe and co-workers;^13^ later, in the year 1972 the Cs_2_PbO_3_ compound was synthesized by Panek *et al.*,^14^ and then latterly the Rb_2_PbO_3_ was again synthesized in 1977 by Hoppe and Stöver.^15^ From these earlier experimental studies, it was found that the K_2_PbO_3_, Rb_2_PbO_3_, and Cs_2_PbO_3_ existed in an orthorhombic structure with space group *Cmc*2_1_ (no. 36). More recently, in 1982, Brazel and Hoppe synthesized the Li_2_PbO_3_ by decomposing K_2_Li_6_[Pb_2_O_8_] and reported a monoclinic structural symmetry (*C*2/*c* space group) for this compound.^16^ The interesting information about the *Cmc*2_1_ symmetry materials of X_2_PbO_3_ is that in these structures, similar to the prototype Na_2_SiO_3_, and Na_2_GeO_3_, the presence of links between oxygen and Pb-atoms led to the formation of a three dimensional network of a tetrahedral chain of [PbO_4_]. The link forms the bridge-oxygen (BO) and non-bridge-oxygen (NBO), where alkali atoms such as X = K, Rb, and Cs are bonded.^17^ Therefore, K_2_PbO_3_ (KPO), Rb_2_PbO_3_ (RPO), and Cs_2_PbO_3_ (CPO) are non-centrosymmetric in structure, and consequently exhibit piezoelectric properties. Moreover, in case of Li_2_PbO_3_ (LPO) and Na_2_PbO_3_ (NPO), since the compounds are centrosymmetric, they do not possess piezoelectricity.

In the past few decades, experimental and theoretical insights into novel multi-function energy materials especially for TE and piezoelectric applications have become a hot topic among researchers.^18–20^ Thermoelectricity and piezoelectricity hold the requisite hallmarks for green energy resources as they produce electricity without any emissions of toxic pollutants. The glass-like materials possessing high mechanical and thermal stability, low lattice thermal conductivity (*κ*_L_), high melting temperatures (*T*_m_), ease of availability, and the cost-effectiveness have made these materials perfect candidates for multi-use energy generators. However, the presence of the toxic Pb-element in our proposed compounds *i.e.*, X_2_PbO_3_ have made this topic more challenging. From various research articles, it has been observed that the toxicity offered by the Pb-element can be successfully minimized, and the prominent strategies for this include chelation therapy, nano-encapsulation, and *N*-acetylcysteine (NAC).^21–23^ A common strategy to reduce toxicity is the substitution of the Pb element with less toxic elements such as Sn and Pb. Since, both elements possess compatible ionic radii and electronic structures, they enable the replacement of Pb without major lattice distortions, while reducing the overall Pb content. Moreover, from a functional perspective, Sn-substitution is particularly attractive for energy-related applications because it retains the narrow band gap and high carrier mobility that are essential for optoelectronic and photocatalytic processes.^24,25^ However, for X_2_PbO_3_ materials, when X moves from Li → Cs, the environmental risk of X_2_PbO_3_ is expected to increase, as larger alkali ions weaken the glass network and facilitate Pb release. To reduce the energy crisis rendered by the global demands for energy sources, thermoelectricity and piezoelectricity could be an innovative approach although their efficiencies are low. From the recent study led by Zosiamliana *et al.*,^26^ the TE efficiencies at *T* = 1200 K for Pb-based perovskites such as PbTiO_3_, PbZrO_3_, and PbHfO_3_ were found to be *ZT* = 0.64, 0.66, and 0.61, revealing the suitability of Pb-based materials for TE devices. Also, recent reports reveal the relevancy of glass-like materials such as Na_2_SiO_3_ and Na_2_GeO_3_ (the prototype compounds for KPO, RPO, and CPO) for piezoelectric devices, with a response of *e*_33_ = 0.22 C m^−2^ and *e*_33_ = 0.91 C m^−2^, respectively.^12,27,28^ Experimentally and theoretically, only few research studies have been carried out on glass-like materials for TE applications. Recently, an efficiency of *ZT* = 0.027 at *T* = 393 K was reported for Na_2_SiO_3_ as a graphite/mixture (CuSO_4_ + CoOH + SiO_2_ + Na_2_SiO_3_)/aluminum by Chira *et al.*^29^ Thus, the unmet research area that needs to be addressed is the piezoelectric and TE applications of X_2_PbO_3_ glass-like materials.

As far as we know, from several literature surveys, only a few experimental and theoretical studies were conducted on these materials. Recently, the photo-catalytic use of these compounds for solar-to-hydrogen conversion was reported by Gelin and colleagues^30^ however, this failed to provide a comprehensive insight into the fundamental properties. As a result, the main focus of this work will be on the thorough analysis of the fundamental characteristics of X_2_PbO_3_ (X = Li, Na, K, Rb, Cs) and their applications for piezoelectric and TE materials employing hybrid-DFT.

## 2 Computational details

In this work we have performed density functional theory (DFT) calculations as implemented in the CRYSTAL17-code to evaluate the physical properties of X_2_PbO_3_. In this part of the DFT framework the crystal orbitals are described by the linear combination of Gaussian-type functions (GTF).^31^ The atomic centers for all the constituent atoms such as X = lithium (Li), sodium (Na), potassium (K), rubidium (Rb), and caesium (Cs), lead (Pb), and oxygen (O) were described by a revised triple-*ζ* valence plus polarization (TZVP) basis set.^32,33^ In this work, four different exchange–correlation functionals were adopted, namely; (1) the Perdew–Burke–Ernzerhof (PBE) generalized gradient approximation (GGA);^34^ global hybrids: (2) Becke 3-parameter Lee–Yang–Parr (B3LYP),^35,36^ and (3) PBE with 25% Fock exchange (PBE0);^37^ and (4) the range-separated hybrid screened-Coulomb (SC) called HSE06.^38^

The expressions for the employed hybrid functionals are:^39^

1 *E*^B3LYP^_XC_ = *E*^LSDA^_XC_ + *a*_0_(*E*^exact^_X_ − *E*^LSDA^_X_) + *a*_X_Δ*E*^B88^_X_ + *a*_C_Δ*E*^PW91^_C_

Here, *a*_0_, *a*_X_, and *a*_C_ are semi-empirical coefficients, *E*^LSDA^_XC_ is the local spin density exchange–correlation, *E*^exact^_X_ is the exact exchange energy, Δ*E*^B88^_X_ is Becke’s gradient correction for exchange, and Δ*E*^PW91^_C_ is the Perdew and Wang gradient correction.2
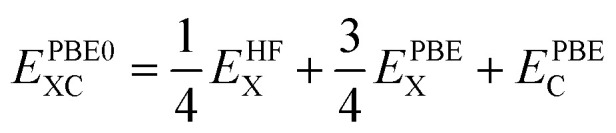
Here, the full PBE correlation energy and the 3 : 1 ratio of PBE to HF exchange energies determine the PBE0 functional.3
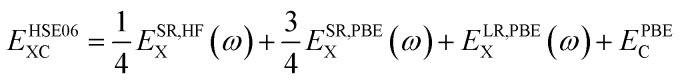
where, *ω* = 0.2 is the screening parameter, *E*^SR,HF^_X_ represents the short-range Hartree–Fock exact exchange functional, *E*^SR,PBE^_X_, and *E*^LR,PBE^_X_ are the short-range, and long-range PBE exchange functionals, respectively, and *E*^PBE^_C_ is the full correlation energy.

For the structural optimizations, an analytical quasi-Newtonian approach combined with Hessian Broyden–Fletcher–Goldfarb–Shanno (BFGS) scheme was used.^40,41^ To check the convergence, the gradient components and nuclear displacements with tolerances on their root-mean-square (RMS) were set to 0.0001 and 0.0004 Hartree (Ha), respectively. The accuracy of the convergence criteria was set to the five thresholds which control the overlap and penetration of the Coulomb integrals; the overlap for HF exchange integrals, and the pseudo-overlap were set to (10^−7^, 10^−7^, 10^−7^, 10^−7^, 10^−7^, 10^−14^). The first Brillouin zone integration was performed using a 10 × 10 × 10 *k*-mesh within the Monkhorst–Pack scheme,^42^ and an energy convergence criteria of 10^−7^ Ha was considered. Then, to ensure that the optimized geometries correspond to true minima on the potential energy surface (PES), we conducted an equation-of-state (EOS) analysis on the optimized structures. The total energy as a function of cell volume was examined to confirm that the optimized configurations correspond to the lowest point (global minimum) on the energy–volume curve, thereby validating the structural stability and energetic consistency of the obtained geometries. For the calculation of the physical properties we have set the higher *k*-mesh of 12 × 12 × 12. For the calculation of the elastic and piezoelectric properties, we have opted for the best suited exchange–correlation functionals depending on the accuracy in reproducing the experimental lattice parameters. To verify the structural stability, we performed an *ab initio* molecular dynamics (AIMD) simulation with an nVT canonical ensemble (calculation details are provided in Section 3.1).^43–45^

For the TE properties calculation, the Boltzmann transport semi-classical equation (BTE) within a rigid band approximation (RBA) using BoltzTraP as implemented in the CRYSTAL17-code was employed.^7,46,47^ The wave functions were recalculated at a dense *k*-mesh of 42 × 42 × 42 within the first Brillouin zone. A constant relaxation time approximation (CRTA) for carriers was assumed for all the investigated compounds, and fixed at *τ* = 10^−14^ s (default *τ* for BoltzTraP-code).

## 3 Results and discussions

### 3.1 Structural properties and stability

**Fig. 1:**
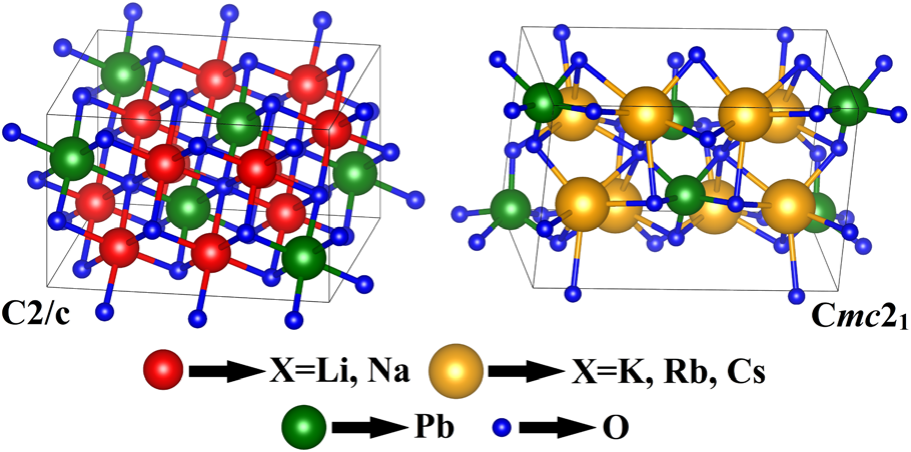
The *C*2/*c* (X = Li, Na), and *Cmc*2_1_ (X = K, Rb, Cs) structures of X_2_PbO_3_ viewed using an external program called VESTA.^48^

**Fig. 2:**
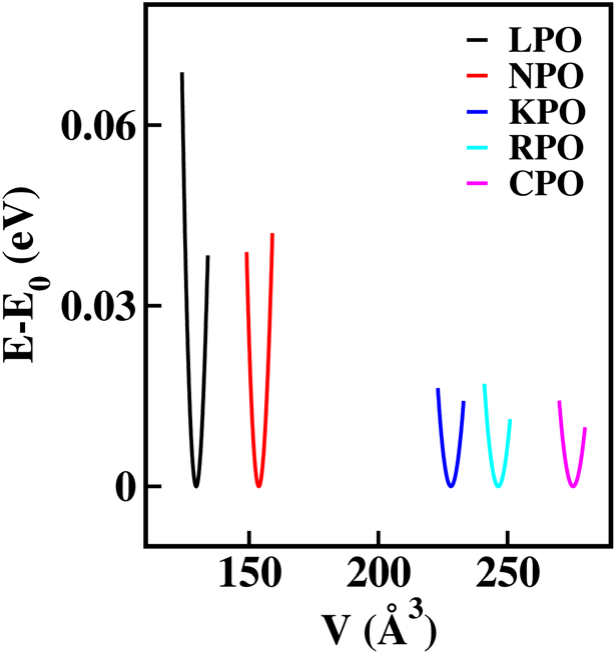
Energy *vs.* volume curves of X_2_PbO_3_ (X = Li, Na, K, Rb, Cs) calculated using the Birch–Murnaghan’s EOS curve fitting method. Here, *E*_0_ is ground state energy.

The structural optimization results using various adopted functionals such as PBE-GGA, B3LYP, HSE06, and PBE0 reveal that the investigated compounds X_2_PbO_3_ crystallized in monoclinic symmetry (*C*2/*c* space group) for X = Li, and Na, and in an orthorhombic structure (*Cmc*2_1_ space group) for X = K, Rb, and Cs [see Fig. 1]. The agreements between the calculated lattice constants and the available experimental data for each compounds are presented in Table S1. For LPO, KPO, RPO, and CPO the global hybrid-PBE0 functional is found to be the most relevant functional in reproducing the experimental data with |Δ*a*|, |Δ*b*|, and |Δ*c*| < 2%, as it is known that the global hybrid-PBE0 functional reproduces better lattice parameters and the electronic band structure of small or large band gap solids. It is important to remain aware that the stability of the studied systems will be impacted if the lattice constant’s precision deviates by more than 2%.^26^ From the formation energy (*E*^f^) calculations using eqn (4), a negative *E*^f^ reveals the ground state structural stabilities for all the considered systems in their corresponding phases, and implies a possible realization of their experimental synthesis. Furthermore, a greater negative *E*^f^ is observed for each compound with the PBE0 functional, suggesting this functional is the most favored to acquire the energy ground state. As a result, each compound’s optical, elastic, and piezoelectric properties are investigated using the PBE0 functional. However, the electronic and TE properties are explicitly calculated using the four different adopted functionals in order to verify the role played by exchange–correlation functions during the study of TE properties.4
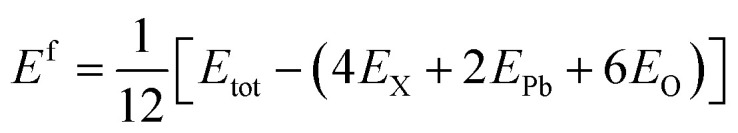
Here, *E*_tot_ is total ground state energy, and *E*_X_, *E*_Pb_, and *E*_O_ are the corresponding single atom ground state energies for the X, Pb, and O atoms. Since there are 12 atoms in the unit cell, the right hand side (RHS) of eqn (4) is divided by 12. To obtain the most stable configurations for all the examined systems, total energies *versus* the unit cell volumes were fitted through the Birch–Murnaghan equation of states (EOS) scheme given by eqn (5) using the PBE0 functional.^49,50^ From the depicted smooth parabolic curves in Fig. 2, the energy difference where *E* − *E*_0_ = 0 eV corresponds to the most stable structure.5
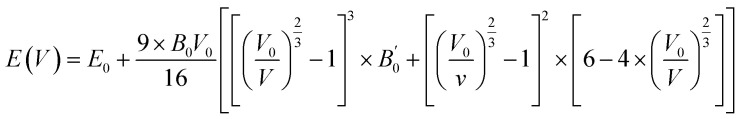


**Fig. 3:**
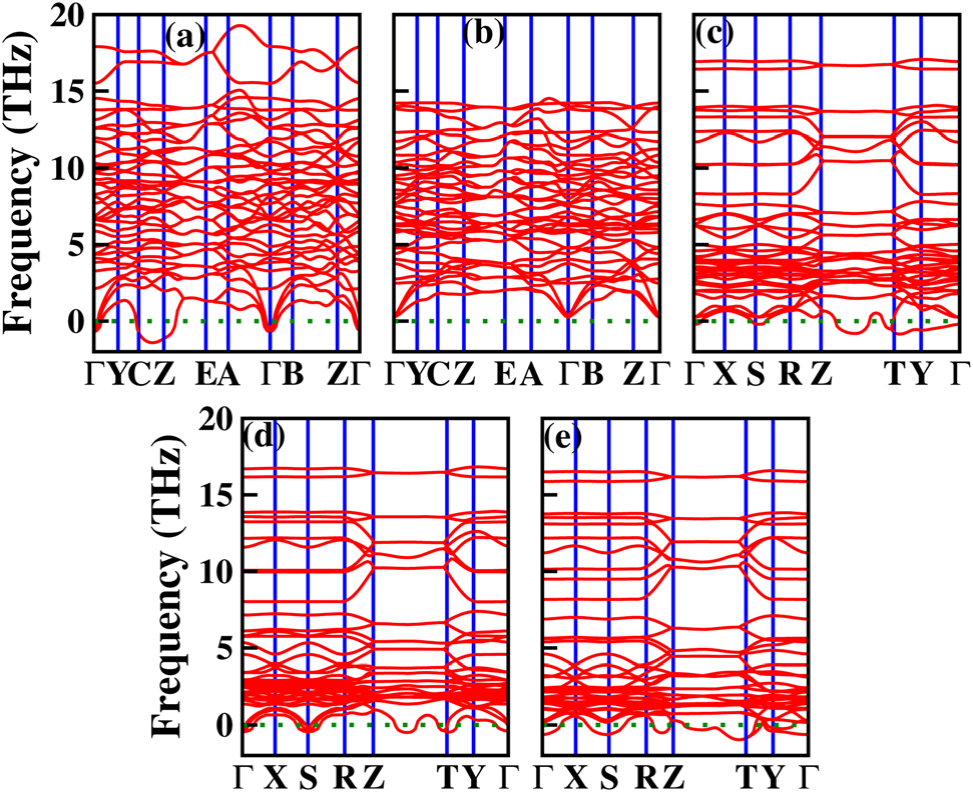
Phonon dispersion curves of X_2_PbO_3_: (a) X = Li, (b) X = Na, (c) X = K, (d) X = Rb, and (e) X = Cs.

**Fig. 4:**
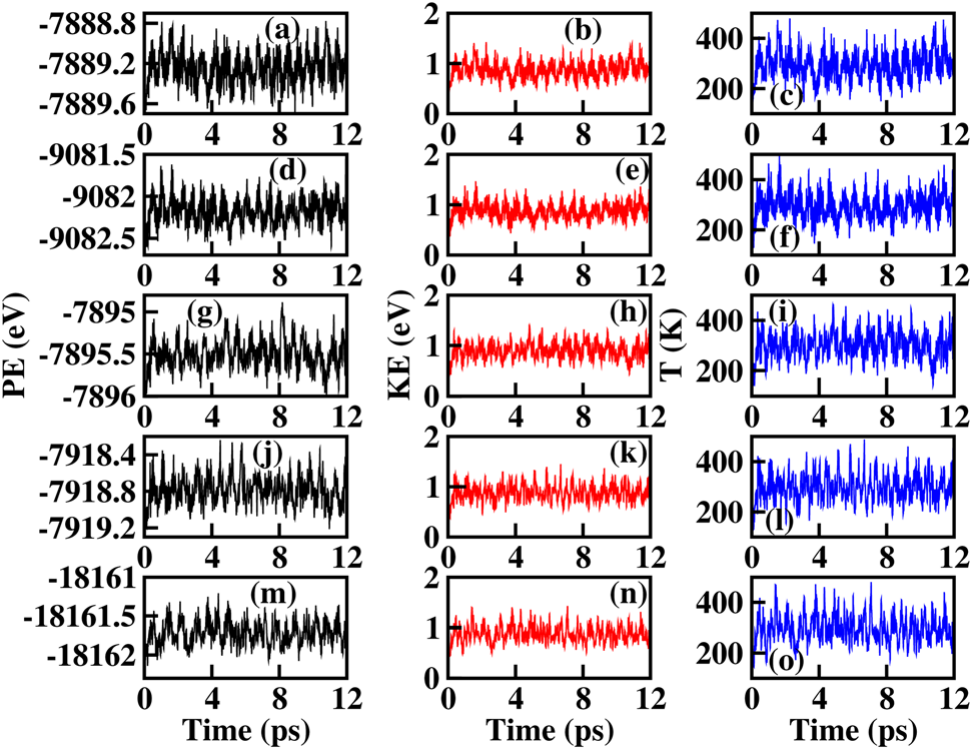
The potential energy (PE), kinetic energy (KE), and evolution of temperature (*T*) as a function of time steps calculated using MD simulations based on a nVT-canonical ensemble: (a–c) X = Li, (d–f) X = Na, (g–i) X = K, (j–l) X = Rb, and (m–o) X = Cs.

To verify the thermal and dynamic stabilities of the studied systems, we have performed molecular dynamics (MD) simulations for each of the relaxed conventional cell structures (*i.e.*, two times the number of atoms in the unit cell) both at room temperature and at *T* = 850 K, and the phonon dispersion curve calculations. Since MD simulations cannot be performed using the CRYSTAL17-code, throughout this computational process the QuantumATK Q-2019.12 code, which relies on a linear combination of the atomic orbital method (LCAO) with the canonical ensemble (nVT) based on Nosé–Hoover thermostat, was adopted.^51–53^ A 12 ps total simulation time with 4 fs time step for room temperature stability and a 5 ps total simulation time for *T* = 850 K were considered during the simulations for all the structures. The graphical representation of the MD simulations (see Fig. 4 and S16) calculated in nVT ensemble where the number of atoms, cell volume, and temperature are constant provides a more realistic insight into the evolution of energies (PE, and KE), and temperature (*T*). Here, PE and KE represent the sum of energies from non-bonded and bonded interactions, and the heat absorbed by the systems, respectively. The higher evolution of *T* with time suggests more movement of the atoms, thereby resulting in more fluctuation of the KE. The nearly linear fluctuations of PE, KE, and *T* even up to 12 ps time steps reveals the thermal stability for all the investigated materials at room temperature and the nearly linear fluctuation of the MD parameters at *T* = 850 K verified the thermal stability at the higher temperature range. To verify the dynamic stability of the studied systems, we have presented the GGA calculation of the lattice dynamics with their relaxed structures. The graphical representation of the phonon dispersion curve within the first Brillouin zone is shown in Fig. 3. Due to the presence of 24 atoms in a conventional cell, we can have 72 vibrational modes of phonons at any *q*-point, consisting of three acoustic modes and 69 optical modes. The absence of imaginary phonon frequencies for NPO confirms the dynamic stability, while for other systems, imaginary phonon frequencies (≤0.25 THz) at some *q*-points, suggested that they are dynamically unstable. However, such low imaginary phonon frequency values could arise from numerical noise during the calculation process. Also, negative phonon frequencies in glass-like materials (the generic nature of glass materials) indicate the presence of soft vibrational modes, consistent with their tendency toward amorphous or disordered configurations. They can also partly arise from computational limitations in modeling inherently non-periodic systems using periodic boundary conditions.

### 3.2 Electronic properties

**Fig. 5:**
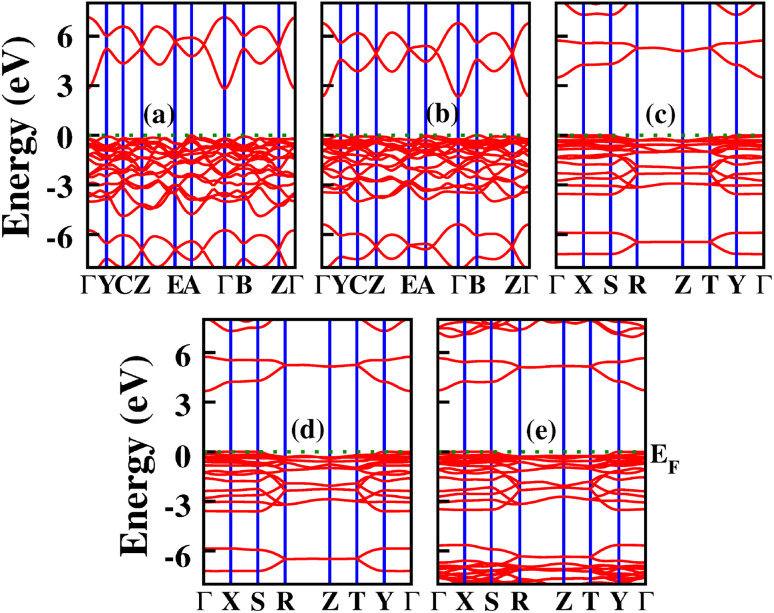
Calculated band structures for X_2_PbO_3_ using the PBE0 functional: (a) X = Li, (b) X = Na, (c) X = K, (d) X = Rb, and (e) X = Cs.

**Fig. 6:**
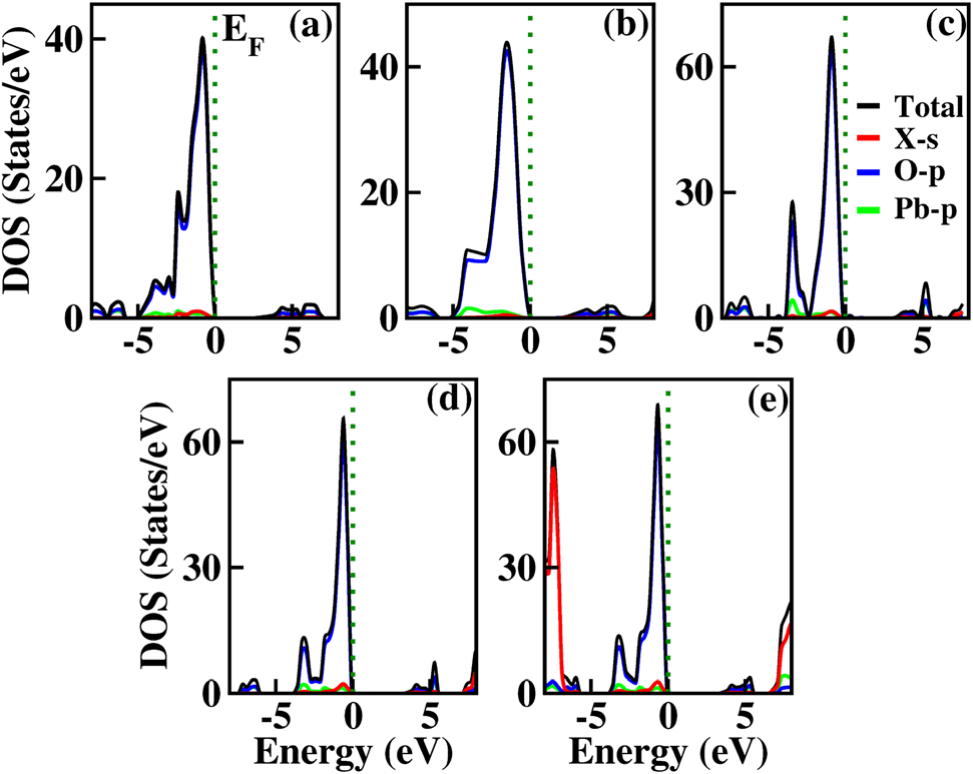
Calculated DOS for X_2_PbO_3_ using the PBE0 functional: (a) X = Li, (b) X = Na, (c) X = K, (d) X = Rb, and (e) X = Cs.

The emergence of physical properties can be understood by interpreting electronic properties with regard to orbital interactions at the atomic level. In this section, we have studied the electronic properties which includes band structures, density of states (DOS), and atomic charge transfer (*Q*_T_) using PBE-GGA, B3LYP, HSE06, and PBE0 functionals. The presented band structures, and DOS calculated using the PBE0 functional in Fig. 5 and 6 (for PBE-GGA, B3LYP, and HSE06 see Fig. S1 and S2), and the energy gap (*E*_g_) at the high symmetry points given in Table 1, revealed the indirect semi-conducting band gap nature for LPO, and NPO with the top and bottom of the valence and conduction bands lying at the *A* and *Γ*-symmetry points. For KPO, RPO, and CPO, *E*_g_ are along the *Γ*-symmetry suggesting a direct band gap semiconductor behavior. The incorporation of hybrid functional flavors during the electronic properties calculations has a significant effect on the *E*_g_ due to the shifts in the energy levels, however, the energy band profiles are preserved. Evidently, from Table 1, since the *C*2/*c*-X_2_PbO_3_ compounds have distinct local coordination environments and Pb–O network conductivities compared to those of the *Cmc*2_1_-X_2_PbO_3_ compounds, in the *C*2/*c*-X_2_PbO_3_ structures, the Pb–O bonds experience stronger octahedral distortion and shorter Pb–O bond lengths, leading to enhanced Pb–O orbital overlap. This stronger hybridization raises the valence band maximum and narrows the *E*_g_. Hence, within the *C*2/*c* structures, the smaller cation X = Li experiences increasing overlap and reducing *E*_g_ compared to X = Na, while in the *Cmc*2_1_-X_2_PbO_3_ structures, there is a less distorted Pb–O framework and more symmetric coordination geometry. They have reduced Pb–O orbital overlap and weakened Pb–O hybridization, which lowers the valence band maximum and increases the *E*_g_. Thus, within *Cmc*2_1_ structures, *E*_g_ increases with increasing ionic radius because the structure becomes more open and less covalently bonded. Comparing the obtained *E*_g_ with the recent report led by Gelin *et al.*,^30^ where *E*_g_ were recorded for the *C*2/*c* symmetry of Li_2_PbO_3_, *P*6_3_/*mmc* symmetry of K_2_PbO_3_, *Pnma* symmetry of Rb_2_PbO_3_, and *Cmc*2_1_ symmetry of Cs_2_PbO_3_ using GGA and GGA+U approximations, one can find that these reported *E*_g_ accord well with our results.

**Table 1:** Electronic band gap (*E*_g_) (in eV), and atomic charge transfer (*Q*_T_) (in |*e*|) using Mulliken population analysis for X_2_PbO_3_ (X = Li, Na, K, Rb, Cs)

X	*E*_g_	*Q* ^T^_X_	*Q* ^T^_Pb_	*Q* ^T^_BO_	*Q* ^T^_NBO_		*E*_g_	*Q* ^T^_X_	*Q* ^T^_Pb_	*Q* ^T^_BO_	*Q* ^T^_NBO_
	**PBE-GGA**						**B3LYP**				
Li	0.88	0.54	1.71	−0.95	−0.92		2.27	0.65	1.99	−1.10	−1.09
Na	0.53	0.74	1.49	−1.01	−0.98		1.83	0.82	1.76	−1.15	−1.12
K	1.23	0.71	1.34	−0.93	−0.92		2.94	0.75	1.55	−1.01	−1.01
Rb	1.45	0.77	1.27	−0.95	−0.93		3.08	0.81	1.47	−1.03	−1.02
Cs	1.53	0.77	1.24	−0.95	−0.92		3.13	0.82	1.43	−1.03	−1.02

	**HSE06**						**PBE0**				
Li	2.23	0.61	2.01	−1.08	−1.07		2.82	0.61	2.04	−1.09	−1.08
Na	1.79	0.81	1.77	−1.15	−1.12		2.34	0.82	1.80	−1.16	−1.13
K	2.84	0.75	1.57	−1.03	−1.03		3.50	0.75	1.59	−1.03	−1.03
Rb	3.02	0.82	1.50	−1.05	−1.04		3.67	0.82	1.51	−1.05	−1.04
Cs	3.09	0.81	1.47	−1.03	−1.02		3.72	0.81	1.48	−1.03	−1.03

Insight into the general distribution of electronic states at different energy levels and the electronic charge distributions upon the formation of bonds are gained through the analysis of DOS and Mulliken population analysis.^54^ From the DOS plots, it is comprehensible that for all the studied compounds, the main contributing states near the Fermi level (*E*_F_) along the valence band region are from the O-p states, while the major contributions along the conduction region are from the complex hybridized states of X-s, Pb-p, and O-p, which are analogous to those closely related glass-like materials such as Li_2_SiO_3_, Na_2_SiO_3_, Li_2_GeO_3_, and Na_2_GeO_3_.^12,27,55,56^ For each compound, from the relatively greater atomic concentrations of NBO than BO, and from the simple altercation of on-site Coulomb repulsion, the NBO-p orbitals which have greater energy (or lower binding energy) are likely to have larger valence charges than BO-p orbitals. Therefore, among the two prominent DOS peaks along the valence region found between −5 eV and 0 eV, the first peak which is closer to the *E*_F_ is mainly from NBO-p contributions, while the second peak which is at the lower energy region is predominantly contributed by BO-p. Furthermore, from the data of the Mulliken population analysis of the relative atoms’ charge distributions calculated from different adopted functionals presented in Table 1, the X and Pb atoms transfer charges while the NBO and BO atoms accumulate them for all the considered systems. By establishing a correlation between the charge transfer and DOS, it is possible to clarify the impact of various functionals on the electronic properties calculation. For LPO, and NPO, from the DOS plots it is possible to notify that the total DOS (TDOS) contribution near *E*_F_ is comparatively higher with the PBE-GGA functional when compared to other adopted functionals. This is mainly due to a significant change in *Q*^T^ between the PBE-GGA and hybrid functionals’ calculated results. With hybrid functionals the charge accumulated by the BO and NBO becomes larger suggesting a lower number of unoccupied states, which subsequently reduces the TDOS contribution around *E*_F_. However, for KPO, RPO, and CPO an opposite phenomenon of the PBE-GGA estimated TDOS exhibiting a lower contribution near *E*_F_ than those hybrid functionals employed is observed, even-though *Q*^T^ for BO and NBO becomes larger with hybrid functionals. The structural arrangements of the materials can provide insight into the reason behind this behavior. The X–BO and X–NBO bond lengths in the *C*2/*c* symmetry compounds (*i.e.*, X = Li and Na) are identical, while they are unequal in the *Cmc*2_1_ symmetry compounds (*i.e.*, X = K, Rb, and Cs). The difference in the structural arrangements of these materials for *C*2/*c* and *Cmc*2_1_ symmetry compounds is the key factor for the non-centrosymmetric nature of the KPO, RPO, and CPO compounds.

Fig. S15 illustrates the two-dimensional differential charge density map for the investigated compounds, providing profound insights into the charge distribution among the X–BO/NBO, Pb–BO/NBO, and X–Pb interactions. The visualization highlights charge depletion in red and charge accumulation in blue, reflecting the redistribution of electron density within the system. This charge rearrangement plays a crucial role in elucidating the relationship between charge transfer mechanisms and electronic band structures, particularly concerning electron transitions from the valence band to the conduction band through the electronic band gap. In each case, X and Pb atoms predominantly act as electron donors, leading to localized charge depletion in their vicinity. In contrast, oxygen atoms, due to their higher electronegativity, attract electron density, resulting in significant charge accumulation around O-sites. This pronounced electron localization at oxygen centers underscores their function as electron-rich species, thereby influencing the overall electronic structure and bonding nature of the studied materials. The observed charge density differences align well with other electronic properties, including the calculated density of states (DOS) and Mulliken population analysis. Furthermore, while no substantial variations are observed between the employed functionals, noticeable differences arise when comparing the *C*2/*c*-X_2_PbO_3_ and *Cmc*2_1_-X_2_PbO_3_ phases. Specifically, the *C*2/*c*-X_2_PbO_3_ structure exhibits a more pronounced presence of red and blue regions than the *Cmc*2_1_-X_2_PbO_3_ phase, indicating stronger charge transfer interactions. This suggests a more significant charge redistribution in the *C*2/*c* system, highlighting its enhanced electronic polarization and bonding characteristics. Also, the analysis of the electronic structure has been expanded to correlate the observed trends with underlying Pb–O bonding and orbital hybridization. The variations in *E*_g_ are attributed to the interplay between Pb–O covalency, cation size, and crystal symmetry, where enhanced Pb–O hybridization in the *C*2/*c* phase narrows the *E*_g_, while reduced overlap in the *Cmc*2_1_ phase widens it.^57^ Charge-density and DOS analyses confirm the dominant Pb-6s/O-2p contributions near the band edges. These electronic features govern carrier transport and suggest that structural or compositional tuning could optimize the X_2_PbO_3_ compounds for thermoelectric, sensor, or optoelectronic applications.^58^

### 3.3 Optical properties

To interpret the interaction between electromagnetic radiation and the studied X_2_PbO_3_ materials, we have explored the optical properties by calculating the complex dielectric constants (*ε*), extinction coefficient, refractive index, reflectivity, and absorption coefficient as a function of photon energy using the PBE0 functional. It is known that at higher frequencies, *ε* is divided into two parts, namely; the real part (*ε*_1_), and the imaginary part (*ε*_2_) [see eqn (6)].^59^

6 *ε* = *ε*_1_ + i*ε*_2_

For calculating other optical parameters like extinction coefficient (*k*), refractive index (*η*), reflectivity (*R*), and absorption coefficient (*α*), the employed formulae are:7
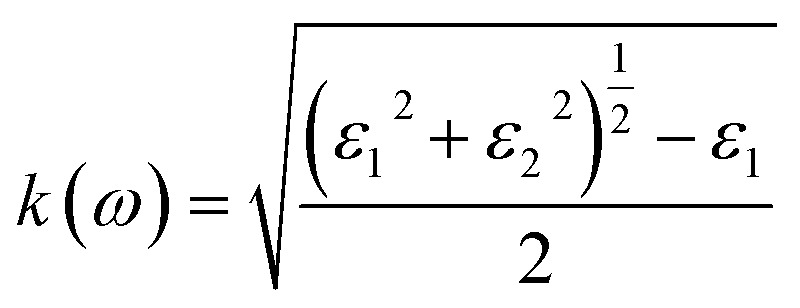
8
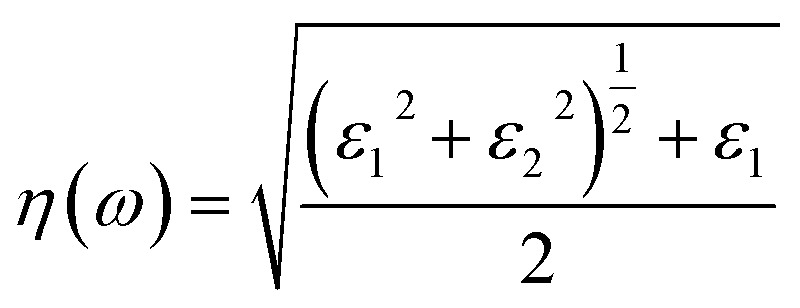
9
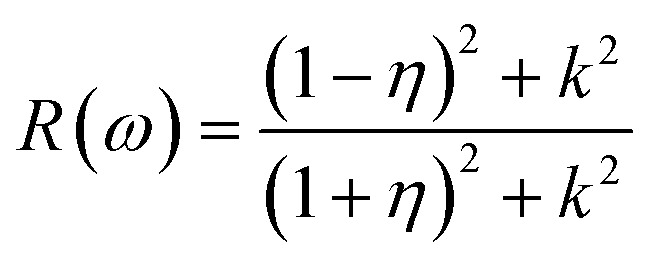
10
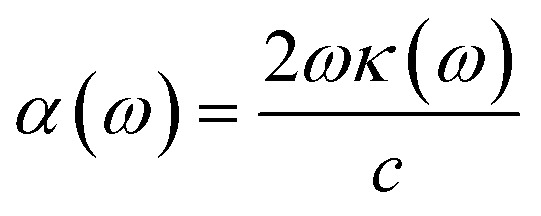


**Fig. 7:**
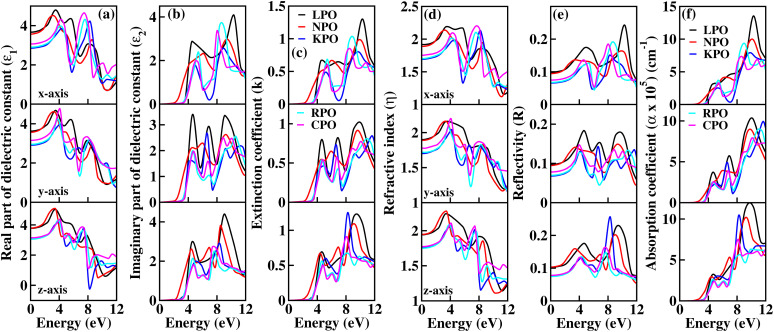
Calculated optical properties of X_2_PbO_3_ (X = Li, Na, K, Rb, Cs) using the PBE0 functional: (a) real part of dielectric constant (*ε*_1_), (b) imaginary part of dielectric constant (*ε*_2_), (c) extinction coefficient (*k*), (d) refractive index (*η*), (e) reflectivity (*R*) and (f) absorption coefficient (*α*).

The *ε*_1_ which is closely interrelated with *η* determines the amount of material polarization and dispersion of electromagnetic radiation when interacting with the material surface [see Fig. 7(a) and (d)]. The calculated static real dielectric constants *ε*_1_(0) are in the order of LPO > NPO > CPO > RPO > KPO, with values in the range of 2.80 to 3.90 arb. unit, for all *x*, *y*, and *z*-axes. The *ε*_1_(0) for the investigated materials are all higher than those of glass-like materials such as Na_2_SiO_3_, whose values is in the range of 1.00 to 1.40 arb. unit, while for Na_2_GeO_3_, *ε*_1_(0) is comparable with X_2_PbO_3_, whose value is ∼3.00 arb. unit.^12,27^ Along the *x*-axis, the first prominent peaks for *ε*^*x*^_1_ are found within 3.0 to 4.5 eV and undergo blue shifting as X goes from Na → Li → Cs → Rb → K. This indicates that the maximum probable interaction with electromagnetic radiation not only differs but also varies across frequency ranges for each compound. Among all the investigated systems, higher static refractive index *η*(0) values are observed for X = Li, and Na than when X = K, Rb, and Cs, with *η*(0) values ranging from 1.5 to 2.0, revealing that they are translucent in nature. Corresponding to *ε*_1_, the curves of *η* show similar trends. The decreasing refractive indices at higher photon energy region *i.e.*, beyond 8 eV, reveals that the electromagnetic radiation no longer has sufficient energy to interact electronically with the material’s electron.

The *ε*_2_ which is closely related to the *k* and *α* corresponds to the inter-band transition between the valence and conduction bands. From Fig. 7(b), on analyzing the direction-wise behavior of *ε*_2_, it is noticeable that *ε*_2_ is highly anisotropic in nature. Clearly, one can find two prominent peaks of *ε*_2_ for each compound along the *x* and *z*-axes, and three distinct peaks in the *y*-axis. Along the *x*-axis, the first prominent peaks of *ε*^*x*^_2_ are blue shifted as X moves from Li → K → Cs → Rb → Na. The extinction coefficient (*k*) which determines the materials capacity to absorb radiation of a specific wavelength is depicted in Fig. 7(c). The obtained minimum threshold energies for X = Li, Na, K, Rb, and Cs are 3.51, 2.33, 3.46, 3.62, and 3.69, respectively, and correspond to their respective optical band gaps. Meanwhile, when compared to other glass-like materials such as Na_2_SiO_3_ (*ε*_2_ = 2.5 eV) and Na_2_GeO_3_ (*ε*_2_ = 3.2 eV), our investigated materials exhibit a higher optical threshold energy. This indicates that these materials are likely to exhibit a stronger response in the higher visible-to-ultraviolet (vis–UV) spectral region. The optical band gaps for X = Na, K, Rb, and Cs, agree well with the first direct electronic transition from the top of the valence band (O-p state, specifically NBO-p state) to the bottom of the conduction band (X-s state) along the *Γ*-symmetry. However, for X = Li the optical band gap is due to the direct electron transition from the third band of the valence band (O-p state) to the bottom of the conduction band (Li-s state) along the Γ-symmetry. Along the *x*-axis, the respective first prominent peaks of *k*^*x*^ for X = Li, Na, K, Rb, and Cs are located within the region 4.50 to 6.00 eV. This corresponds to the first inter-band transition between the valence band and conduction band along the A-symmetry point for LPO, and NPO, and for KPO, RPO, and CPO, the transition is along the Z-symmetry.

The main motive for exploring the optical properties is to determine the reflectivity and the optical absorption [see Fig. 7(e) and (f)]. From the reflectivity spectra as a function of the photon energy, similar to the other optical property parameters, a highly anisotropic trending of the reflectivity curves is found for each compound. The static reflectivity (*R*(0)) values being lower than 0.1 along *x*, *y*, and *z*-axes reveals the semi-conducting behavior of the considered systems. Between 0 and 4.0 eV photon energy, each compound shows increasing reflectivity with energy and then beyond 4.0 eV, drastically fluctuating reflectivity profiles are observed. Since the maximum reflectivity even at higher photon energy regions (*i.e.*, within the ultra-violet region) is less than 25%, it indicates that the examined materials should be good high energy electromagnetic radiation absorbers rather than reflectors. From the curve of the absorption coefficient as a function of photon energy which is consistent with other optical properties such as *ε*_2_ and *k*, one can observe a rapid increase of absorption from ∼3.0 eV photon energy. For each compound, along the *x*, *y*, and *z*-axes the first promising optical absorptions are in the region of 4.0 to 5.0 eV. Since the most active optical absorptions for all the studied materials fall within the UV-region with *α* > 1 × 10^5^ cm^−1^, these compounds might be a potential candidates for optical materials that can serve as UV-radiation shielding materials.

### 3.4 Elastic properties

For practical applicability particularly in the field of piezoelectric and TE applications, the crystalline materials’ strength which is interpreted from the elastic properties plays a key role. In this regard, we report the elastic constant (*C*_*ij*_) and other mechanical properties of X_2_PbO_3_. Since the investigated compounds exist in a monoclinic phase for X = Li, and Na, and orthorhombic phase for X = K, Rb, and Cs, we have ten elastic constants [see Table 2]. The necessary and sufficient conditions for the studied systems to become mechanically stable called Born criteria are given below:^60,61^

**Table 2:** Calculated elastic constants *C*_*ij*_ (in GPa) of X_2_PbO_3_ (X = Li, Na, K, Rb, Cs) using the PBE0 functional

X	*C*_11_	*C*_22_	*C*_33_	*C*_44_	*C*_55_	*C*_66_	*C*_12_	*C*_13_	*C*_23_	*C*_46_
Li	155.99	202.51	230.62	67.19	47.69	54.42	50.38	34.38	67.17	−11.63
Na	136.52	170.85	183.30	70.52	49.14	59.95	46.12	40.96	59.79	8.06
K	83.28	57.12	131.37	25.30	9.52	16.44	32.04	13.19	31.84	0.00
Rb	74.45	63.81	117.98	26.76	7.17	13.32	31.59	11.35	31.19	0.00
Cs	74.09	62.86	102.86	26.99	2.52	13.78	30.15	16.55	26.87	0.00

For the monoclinic phase, the criteria are:11
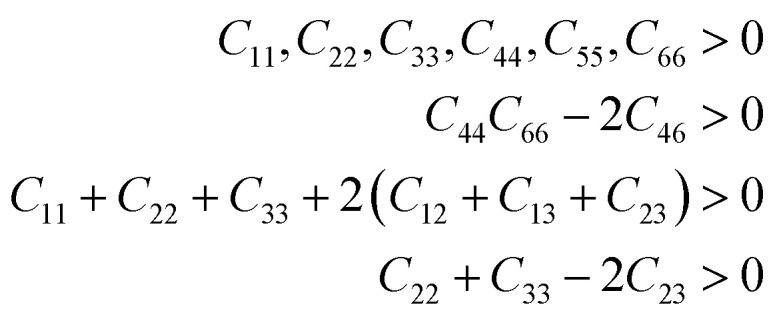


For the orthorhombic phase, the criteria are:12
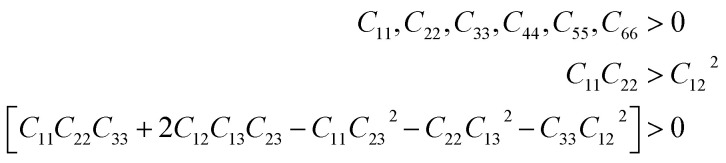


Since the calculated elastic constants *C*_*ij*_ of the X_2_PbO_3_ compounds meet the above mentioned stability criteria, they are mechanically stable. In Table 2, the *C*_11_, *C*_22_, and *C*_33_ determine the system’s stiffness in relation to fundamental stresses while *C*_44_, *C*_55_, and *C*_66_ give their resistance against shear deformation. From the constants *C*_11_, *C*_22_, and *C*_33_ ≫ *C*_44_, *C*_55_, and *C*_66_ it can be understood that the compounds X_2_PbO_3_ tend towards greater resistance to axial compression than shear deformation, which is re-confirmed by the fact that bulk moduli (*B*) are greater than shear moduli (*G*). Also, the high dissimilarity values of *C*_*ij*_ reveals that each compound possesses anisotropic single-crystal elastic behavior. Additionally, our results for the elastic constants also suggest that among the investigated X_2_PbO_3_ materials, the *C*2/*c*-X_2_PbO_3_ structured compounds show greater anisotropic elastic nature compared to those with the *Cmc*2_1_-X_2_PbO_3_ structure. Herein, the elastic moduli: the bulk modulus (*B*), Young’s modulus (*Y*) and shear modulus (*G*) given in Table 3 are determined in terms of Voigt, Reuss, and Hill assumptions, which measure the uniform strain, uniform stress, and their average, respectively.^62–64^ The larger atomic size of X as it goes from Li → Na for the *C*2/*c* structure and from K → Rb → Cs for the *Cmc*2_1_ structure reduces the incompressibility and resistance to volume changes due to the increasing lattice parameters which subsequently increases the inter-atomic distances, and consequently, causes *B* to reduce. In a similar vein, the resulting *Y* and *G* likewise decrease correspondingly. This implies that the studied compounds are soft, flexible, or easier to stretch as X moves down the group. Analyzing the brittleness or ductility based on calculations of Poisson’s (*ν*) (estimated using Voigt, Reuss, and Hill assumptions) and Pugh’s ratios (*k*) (estimated by Hill assumption only) evaluated using eqn (13) and tabulated in Tables 3 and 4, indicates that the *C*2/*c*-X_2_PbO_3_ structured compounds are brittle in nature, while the *Cmc*2_1_-X_2_PbO_3_ structured compounds are ductile. The ductility behavior is in the order of CPO > RPO > KPO > (*k* = 1.75 or *ν* = 0.28) > LPO > NPO, where *k* = 1.75 and *ν* = 0.28 are the critical values for Pugh’s ratio and Poisson’s ratio such that the materials become ductile.13
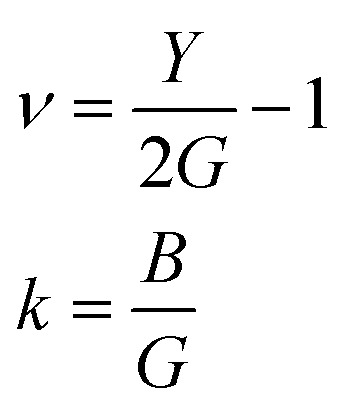


**Table 3:** Elastic moduli (bulk modulus (*B*), Young’s modulus (*Y*), and shear modulus (*G*) all in GPa units), and Poisson’s ratio (*ν*) (unitless) using the PBE0 functional. Here, the subscripts V, R and, H represent Voigt, Reuss and Hill assumptions, respectively

X	*B*_V_	*B*_R_	*B*_H_	*Y*_V_	*Y*_R_	*Y*_H_	*G*_V_	*G*_R_	*G*_H_	*ν*_V_	*ν*_R_	*ν*_H_
Li	99.22	87.11	93.17	156	132.71	144.36	63.01	53.25	58.13	0.24	0.25	0.24
Na	87.16	80.48	83.82	144.10	134.08	139.09	58.84	54.85	56.84	0.22	0.22	0.22
K	47.32	44.56	45.94	59.89	45.85	52.95	23.23	17.26	20.24	0.29	0.33	0.31
Rb	44.95	43.63	44.29	55.83	40.16	48.14	21.59	14.91	18.25	0.29	0.35	0.32
Cs	42.98	42.26	42.62	51.37	23.86	38.14	19.74	8.49	14.11	0.30	0.4	0.35

**Table 4:** Pugh’s ratio (*k*) (unitless) in Hill’s approximation, velocity of sound (*v*) (in km s^−1^), density (*ρ*) (in g cm^−3^), Kleinman coefficient (*ζ*) (unitless), anisotropic factor (*A*_an_) (unitless), machinable factor (*µ*_m_) (unitless), melting temperature (*T*_m_) (in K), Debye temperature (*Θ*) (in K), and Frantsevich ratio (*G*/*B*). Here, the subscripts t, l and av represent transverse, longitudinal and average velocities, respectively

X	*k*_H_	*v*_t_	*v*_l_	*v*_av_	*ρ*	*ζ*	*A*_an_	*µ*_m_	*T*_m_ ± 300	*Θ*	*G*/*B*
Li	1.60	2.89	4.86	3.20	6.94	0.47	1.96	1.39	1475.06	432.07	0.62
Na	1.47	2.95	4.94	3.26	6.53	0.48	2.29	1.19	1359.97	415.51	0.65
K	2.27	2.04	3.87	2.28	4.87	0.52	1.77	1.82	1045.27	254.71	0.43
Rb	2.43	1.78	3.46	1.99	5.74	0.56	1.75	1.65	993.07	216.38	0.41
Cs	3.02	1.50	3.12	1.69	6.29	0.54	2.06	1.58	990.95	177.17	0.33

The information about internal deformation stability and anisotropic factors can be gained through the Kleinman coefficient (*ζ*) and elastic anisotropic factor (*A*_an_) calculated using eqn (14) and presented in Table 4.^65,66^ The *ζ* which is in the range 0 ≤ *ζ* ≤ 1 represents stretching and bending of bonds; a value of *ζ* closer to 1 indicates a negligible contribution of bond stretching. Clearly, for *C*2/*c*-X_2_PbO_3_ structured compounds, the mechanical strength is mostly contributed by bond stretching, while for *Cmc*2_1_-X_2_PbO_3_ structured compounds it is mainly due to the contribution from bending of bonds. Since the obtained *A*_an_ is larger than 1 for all the considered systems, they are highly anisotropic in nature (*A*_an_ = 1 represents isotropic).14
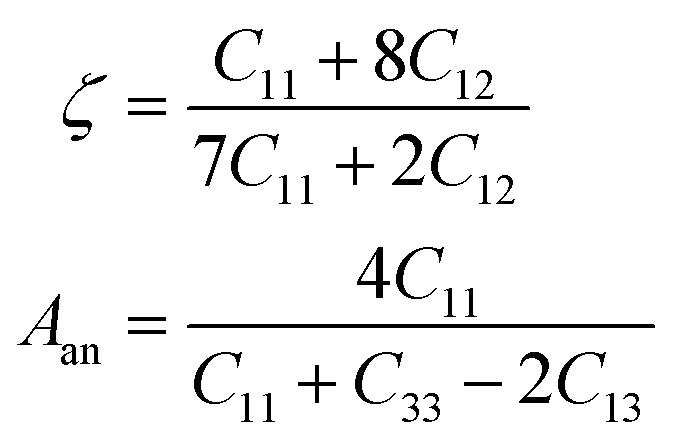


In this novel work, the main requirement for calculating elastic properties is to check the material’s average sound velocity (*v*_av_) determined from the transverse and longitudinal velocities (*v*_t_ and *v*_l_), and the Debye temperature (*Θ*) calculated using eqn (15) and (16):^67–69^15
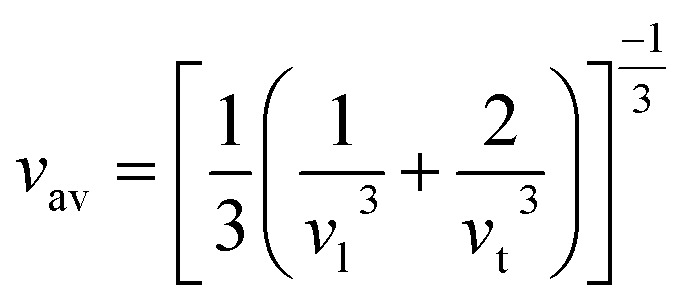
where, 
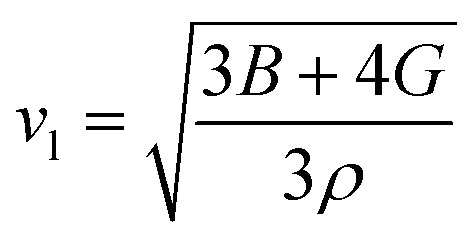
 and 
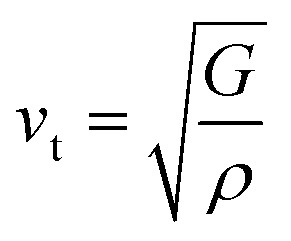
. Here, *ρ* is density.16
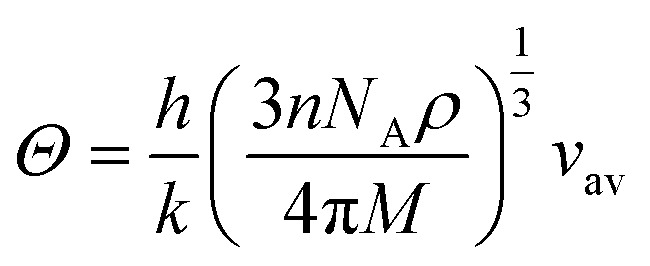
where, *h* is the Planck’s constant, *k* the Boltzmann constant, *n* the number of atoms per formula unit, *N*_A_ is Avogadro’s number and *M* the molecular mass.

From the results presented in Table 4, we observe a decreasing *v*_av_ and *Θ* when X moves from Li → Na → K → Rb → Cs which is mainly due to the increasing atomic masses. For the *Cmc*2_1_-X_2_PbO_3_ structures, the reported *v*_av_ are lower than those analogous compounds such as Na_2_SiO_3_, and Na_2_GeO_3_ with *v*_av_ = 4.02 km s^−1^ and 3.19 km s^−1^, respectively.^28^ As sound velocities are inversely proportional to the density [see eqn (15)], the larger values of *ρ* for the investigated compounds have resulted in the slower *v*_av_ when compared with the Na_2_SiO_3_ and Na_2_GeO_3_ glass-like materials.^12,27,28^ The Debye temperature being higher for the *C*2/*c*-X_2_PbO_3_ structures than the *Cmc*2_1_-X_2_PbO_3_ structures suggests a greater number of active phonon modes in the *C*2/*c*-X_2_PbO_3_.

For the application of any compounds in practice, particularly in the area of piezoelectric and TE applications, machinable factor (*µ*_m_), Frantsevich ratio (*G*/*B*), and melting temperature (*T*_m_) play a key role.^70^ The *µ*_m_ calculated by employing eqn (17) suggests that the investigated X_2_PbO_3_ compounds exhibit an acceptable level of machinability with lower feed forces and intermediate lubricating properties which makes them a potential candidate for piezoelectric materials. The high *T*_m_ and low Frantsevich ratio also reveal that these compounds could be a future TE materials which can be utilized at high temperatures.17
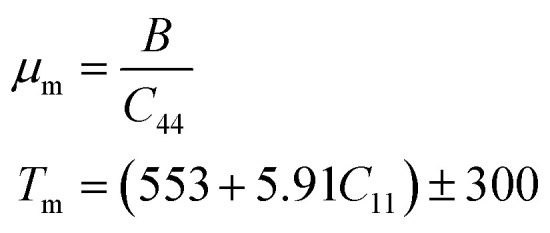


### 3.5 Thermodynamic properties

Knowledge of the state of any system in terms of their energy can be gained through thermodynamic properties calculations. In this regard, we have reported the properties for X_2_PbO_3_ such as the constant volume specific heat (*C*_V_), change in vibrational internal energy (*E*_vib_), vibrational entropy (*S*_vib_), vibrational Helmholtz free energy (*A*_vib_), and the linear thermal expansion coefficient (*α*_L_) as a function of temperature based on the quasi-harmonic Debye model (eqn (18)).^71–74^ From this model,18
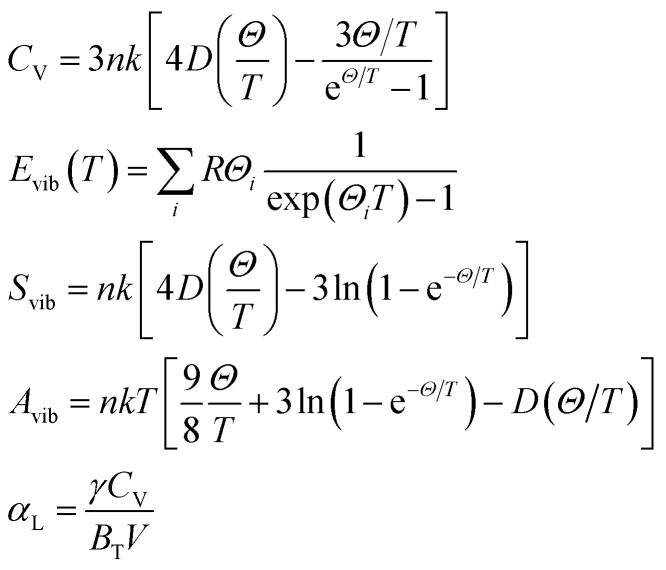
Here, *D* is the Debye integral, *γ* Gruneisen parameter,^75^ and *B*_T_ isothermal bulk modulus defined as,19
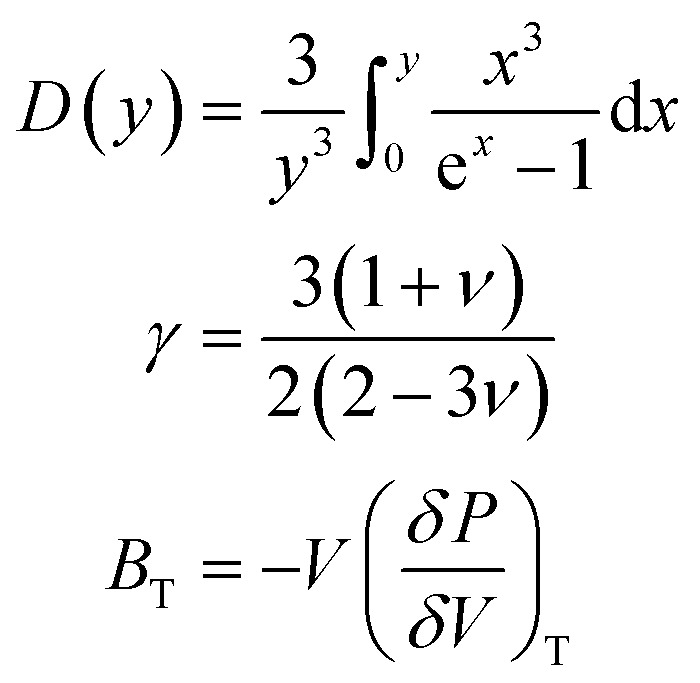
where, *ν* is the Poisson’s ratio.

**Fig. 8:**
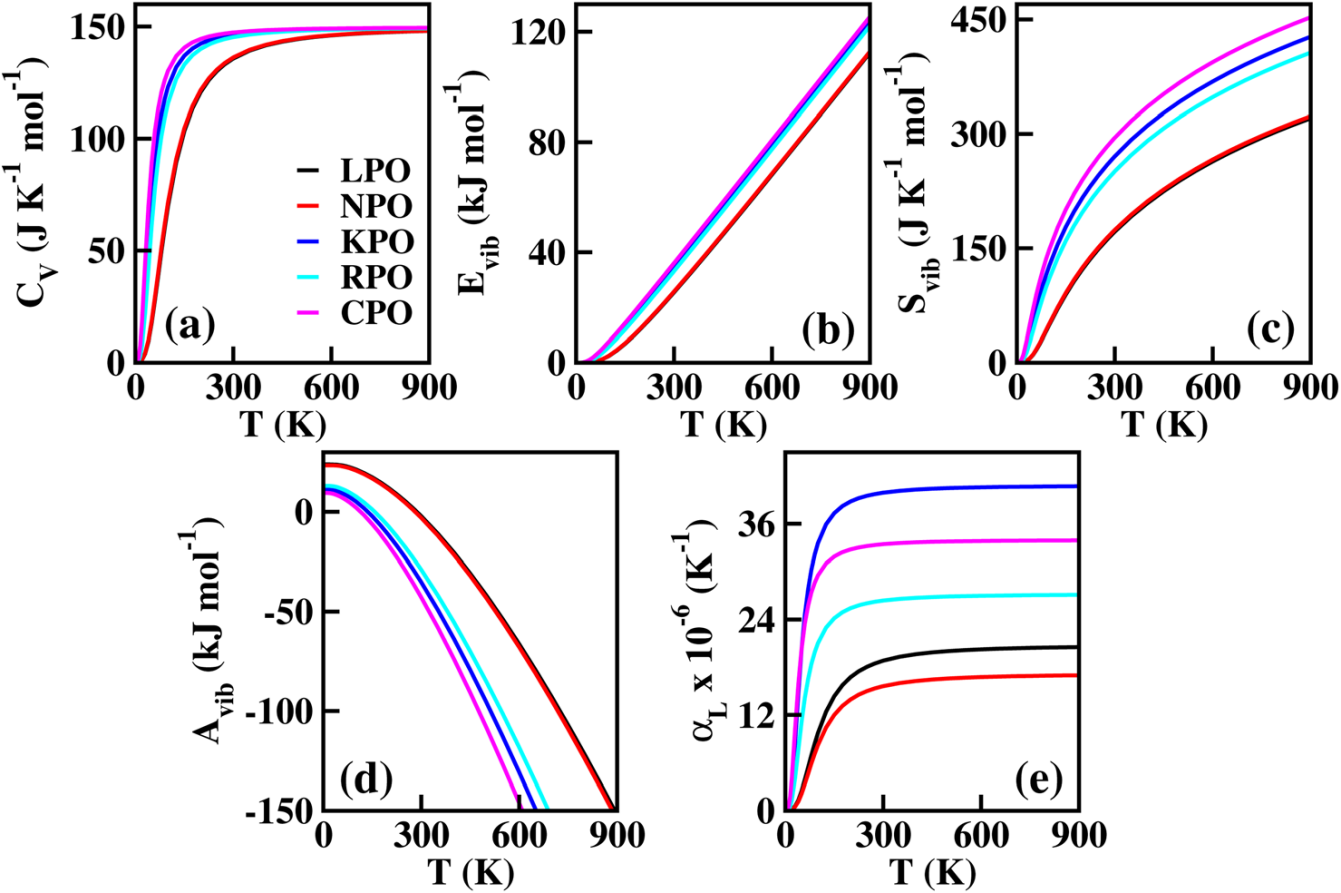
Calculated thermodynamic properties using GGA formalism: (a) constant-volume heat capacity *C*_V_ (J K^−1^ mol^−1^), (b) change in vibrational internal energy *E*_vib_ (kJ mol^−1^), (c) vibrational entropy *S*_vib_ (J K^−1^ mol^−1^), (d) vibrational Helmholtz free energy *A*_vib_ (kJ mol^−1^), and (e) linear thermal expansion coefficient *α* (K^−1^) as a function of temperature.

The intrinsic disorderliness which provides the degree of randomness within the studied systems can be determined from the study of their vibrational entropy (*S*_vib_). From Fig. 8(c), it can be seen that the *C*2/*c*-X_2_PbO_3_ compounds have lower *S*_vib_ than those compounds with *Cmc*2_1_-X_2_PbO_3_ structures. This reveals that in *Cmc*2_1_-X_2_PbO_3_ compounds, the amounts of thermodynamic potential (thermal energy) available for doing useful work are higher than in the *C*2/*c*-X_2_PbO_3_ compounds. The deviation in *S*_vib_ between the monoclinic and orthorhombic structures of X_2_PbO_3_ is mainly due to the variation in the structural and atomic arrangement, and the change in the X-atom component. Also, the increasing *S*_vib_ for each compound with *T* suggests more vibrational states become available or accessible when the temperature escalates. In *C*2/*c*-X_2_PbO_3_ compounds, since the arrangement of atoms especially the bonding between alkali atoms with BO and NBO are identical, nearly equivalent *S*_vib_ curves are obtained. Whereas, for the *Cmc*2_1_-X_2_PbO_3_ compounds, the X bonding with BO and NBO are in different arrangements which leads to different internal energy contributions, thus showing distinct thermodynamic properties. Additionally, the values of *A*_vib_ [see Fig. 8(d)] for the *Cmc*2_1_-X_2_PbO_3_ compounds being lower than those of the *C*2/*c*-X_2_PbO_3_ compounds re-confirms the higher availability of thermal energy for doing useful work in the *Cmc*2_1_-X_2_PbO_3_ structures. In Fig. 8(b), a linearly increasing *E*_vib_ with *T* for both the *C*2/*c* and *Cmc*2_1_-X_2_PbO_3_ structured compounds are observed. This is due to the gradual rising of atoms’ kinetic energy with the continuously increasing heat, which leads to more atomic vibrations. This subsequently results in a continuous escalation of vibrational internal energy.

Comprehension of the lattice vibrational characteristics comes from the study of specific heat (*C*_V_) which is one of the focal points for thermodynamic properties’ investigation. The *C*_V_ curves as a function of temperature for X_2_PbO_3_ materials are presented in Fig. 8(a). Evidently, the curves of *C*_V_ showed similar trending to the nature of other thermodynamic properties parameters plots’ mentioned above. The higher value of *C*_V_ for *Cmc*2_1_-X_2_PbO_3_ structures reveals more heat can be stored with a small gradation in the temperature compared to the same for *C*2/*c*-X_2_PbO_3_ structures. At low temperatures (*T* ≪ *Θ*), the *C*_V_ curves for each compound vary proportionally to *T*^3^, *i.e.*, it follows the Debye’s *T*^3^ law [see eqn (20)],20
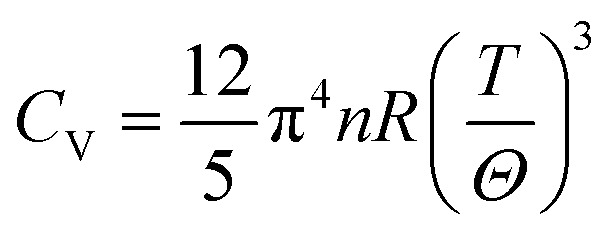


However, at high temperatures (*T* ≫ *Θ*), the *C*_V_ tends to Dulong–Petit limit [see eqn (21)],

21 *C*_V_ ≃ 3*nR*

where *R* = 8.314 J K^−1^ mol ^−1^ is the universal gas constant.

An analysis of the linear thermal expansion coefficient (*α*_L_) which is closely related to the Young’s modulus estimated in the Voigt assumption (uniform strain assumption), yields information about the relationship between the strain that any material endures as a temperature varies. Evidently, from the *α*_L_ plot presented in Fig. 8(e), one can find that the expansion experienced by the *C*2/*c*-X_2_PbO_3_ compounds is nearly twofold to threefold lower than that of the *Cmc*2_1_-X_2_PbO_3_ compounds. The fact that the Young’s modulus is lower in *Cmc*2_1_-X_2_PbO_3_ compounds than *C*2/*c*-X_2_PbO_3_ compounds is a key factor for obtaining higher *α*_L_ for *Cmc*2_1_-X_2_PbO_3_ structures. A comparison of the thermodynamic parameters (*S*_vib_, *C*_V_ and *α*_L_) for the investigated compounds with some other glass-like materials such as Na_2_SiO_3_ and Na_2_GeO_3_ is given in Table 5. Clearly, the investigated compounds are thermally more stable at room temperature since they exhibit higher *S*_vib_ and *C*_V_ than those of the Na_2_SiO_3_ and Na_2_GeO_3_ glass-like materials. Also, the lower expansion coefficient of X_2_PbO_3_ than Na_2_GeO_3_ reveals that the continuous increasing heat will have a reduced effect on the studied X_2_PbO_3_ when compared with Na_2_GeO_3_.

**Table 5:** Comparison table of *S*_vib_ (in J K^−1^ mol^−1^), *C*_V_ (in J K^−1^ mol^−1^), and *α*_L_ (in K^−1^) at *T* = 300 K for X_2_PbO_3_ (current work), Na_2_SiO_3_ and Na_2_GeO_3_

Compounds	*S*_vib_	*C*_V_	*α*_L_
**This work**			
*C*2/*c*-Li_2_PbO_3_	167.26	134.59	18.55 × 10^−6^
*C*2/*c*-Na_2_PbO_3_	170.72	135.77	15.35 × 10^−6^
*Cmc*2_1_-K_2_PbO_3_	265.76	145.77	39.59 × 10^−6^
*Cmc*2_1_-Rb_2_PbO_3_	248.48	144.59	26.21 × 10^−6^
*Cmc*2_1_-Cs_2_PbO_3_	291.68	146.35	33.18 × 10^−6^

**Other’s work**			
*Cmc*2_1_-Na_2_SiO_3_ (ref. 76)	112.50	109.50	—
*Cmc*2_1_-Na_2_GeO_3_ (ref. 12)	163.50	133.90	2.25 × 10^−5^

### 3.6 Piezoelectric and electromechanical coupling properties

As a green method of energy conversion, piezoelectricity has attracted much attention among researchers. Piezoelectric materials may prove to be a viable and sustainable source of energy if an efficient device of high mechanical stress to atomic scale polarization can be discovered. As mentioned in Sections 1 and 3.2, due to the centrosymmetric nature of *C*2/*c*-X_2_PbO_3_ structures, the compounds LPO, and NPO do not possess piezoelectricity. Therefore, in this section, we will be focusing on the piezoelectric properties for the *Cmc*2_1_-X_2_PbO_3_ materials, calculated based on PBE0 functionals, only *i.e.*, the most relevant functional in reproducing experimental lattice parameters for the investigated systems. In this work, piezoelectric tensors are computed using two different methodologies. Firstly, the direct and converse piezoelectric tensors are estimated through the numerical Berry phase (BP) approach which rely on the modern theory of polarization.^77^ According to BP approach [see eqn (22)],22
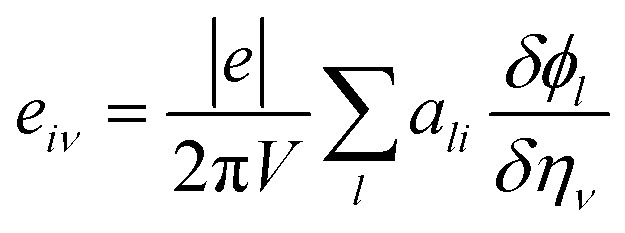
Here, *e*_*iν*_ is the direct piezoelectric tensor, *e* electron charge, *V* volume, *a*_*li*_ is the *i*^th^ Cartesian component of the *l*^th^ direct lattice basis vector *a*_*l*_, *ϕ*_*l*_ is numerical first derivatives of the BP, and *η*_*ν*_ the strain tensor. Also, *i* = *x*, *y*, *z*; *ν* = 1, 2, 3, 4, 5, 6 (1 = *xx*, 2 = *yy*, 3 = *zz*, 4 = *yz*, 5 = *xz*, 6 = *xy*).

The direct and converse piezoelectric tensors are again related by the formula [see eqn (23)]:23
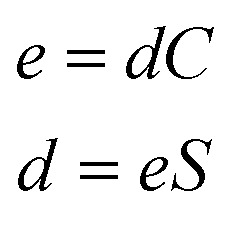


Secondly, the piezoelectric tensors are computed through an analytical approach based on the Coupled Perturbed Hartree–Fock/Kohn–Sham (CPHF/KS) scheme.^78,79^ Herein, in addition to the electronic term’s analytical computation, the internal-strain tensor is used to assess the nuclear-relaxation contribution instead of atomic coordinates’ numerical geometry optimizations at strained configurations. The employed equation [see eqn (24)] for determining the nuclear-relaxation contribution is:24
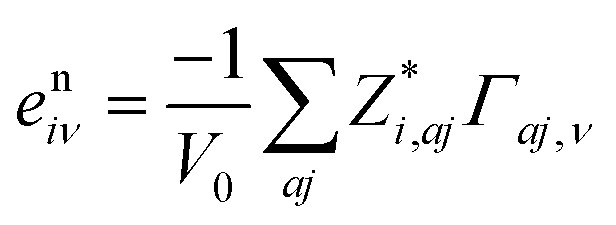
Here, *Γ*_*aj*,*ν*_ and Z* are the displacement-response internal-strain tensor which describes first order atomic displacements as induced by a first order strain, and the tensors containing the dynamic Born effective charges, respectively [see eqn (25)]:25
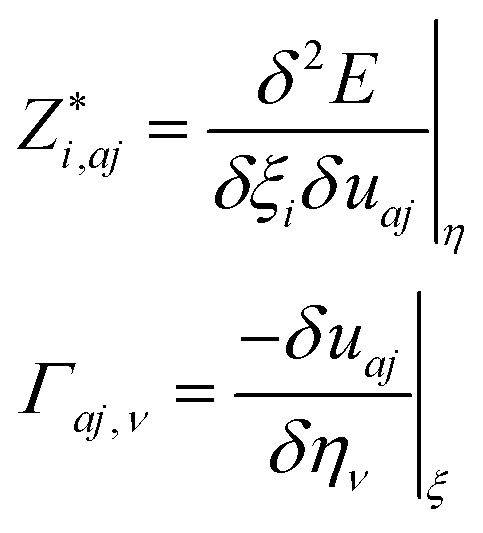
where *u*_*aj*_ are Cartesian components of the displacement vector *u*_*a*_ of atom *a* (*j* = *x*, *y*, *z*).

The calculated piezoelectric tensors for *Cmc*2_1_-X_2_PbO_3_ materials are presented in Table 6 for the BP numerical approach and Table 7 for the CPHF/KS analytical approach. The direct (*e*_*iν*_) and converse (*d*_*iν*_) piezoelectricity presented in Table 6 determine the change of polarization under a finite strain and the strain induced by an applied electric field. At first glance, it can be noticed that from Table 6, the maximum direct piezoelectric constant from BP approach reduces as X goes from K → Cs (magnitude only). The increasing atomic masses of X-atoms when going from K → Cs progressively reduces the distortion of atomic positions from their equilibrium positions, resulting in the dis-amplification of polarization tensor which reduces the piezoelectric constants. For X = K and Rb the maximum responses are *e*_32_ = −0.51, and 0.45 C m^−2^ with the response direction along *z*-axis due to strain *η*_*yy*_. Here, the negative sign (*i.e.*, for X = K) indicates a compressive strain that leads *η*_*yy*_ to a negative value. For X = Cs the maximum response is *e*_33_ = 0.16 C m^−2^, which is in the *z*-axis direction and due to *η*_*zz*_ strain.

**Table 6:** Total direct (*e*_*iν*_ in C m^−2^ unit) and converse (*d*_*iν*_ in pm V^−1^ unit) piezoelectric properties of X_2_PbO_3_ (X = K, Rb, Cs) using the PBE0 functional computed based on a Berry-phase numerical approach

X	Direct					Converse				
	*e*_31_	*e*_32_	*e*_33_	*e*_24_	*e*_15_	*d*_31_	*d*_32_	*d*_33_	*d*_24_	*d*_15_
K	0.32	−0.51	0.30	−0.29	0.29	9.70	−17.41	5.49	−11.53	30.90
Rb	−0.38	0.45	−0.28	0.20	−0.18	−10.63	14.86	−5.24	7.55	−24.71
Cs	−0.01	−0.03	0.16	0.02	0.00	−0.11	−1.13	1.83	0.59	0.08

The electronic term (*e*^e^_*iν*_), nuclear term (*e*^n^_*iν*_), total direct ‘proper’ piezoelectric constant (*e*_*iν*_ = *e*^e^_*iν*_ + *e*^n^_*iν*_) (all in C m^−2^ unit), and electromechanical (EM) coupling factor (*k*_*iν*_) for X_2_PbO_3_ (X = K, Rb, Cs) using the PBE0 functional computed using the CPHF/KS analytical approach

**Table:** 

X	Electronic term					Nuclear term				
	*e* ^e^_31_	*e* ^e^_32_	*e* ^e^_33_	*e* ^e^_24_	*e* ^e^_15_	*e* ^n^_31_	*e* ^n^_32_	*e* ^n^_33_	*e* ^n^_24_	*e* ^n^_15_
K	−0.02	0.10	−0.08	0.14	−0.05	0.36	−0.62	0.38	−0.43	0.34
Rb	0.02	−0.09	0.06	−0.11	0.05	−0.39	0.53	−0.34	0.31	−0.23
Cs	−0.01	0.03	−0.02	0.03	−0.02	0.28	−0.08	0.61	−0.03	0.00

**Table:** 

X	Total					EM coupling				
	*e*_31_	*e*_32_	*e*_33_	*e*_24_	*e*_15_	*k*_31_	*k*_32_	*k*_33_	*k*_24_	*k*_15_
K	0.34	−0.52	0.30	−0.29	0.30	0.41	0.63	0.36	0.36	0.39
Rb	−0.37	0.44	−0.28	0.20	−0.19	0.44	0.52	0.33	0.24	0.23
Cs	0.27	−0.05	0.60	0.01	−0.02	0.31	0.06	0.69	0.01	0.02

It is well-known that performing piezoelectric properties calculations in an analytical fashion using the internal-strain tensor of energy second-derivatives with respect to atomic displacements and lattice deformations in combination with the inter-atomic force constant Hessian matrix, *i.e.* CPHF/KS, approach shows better accuracy than performing the calculation through numerical geometry optimizations to relax atomic positions at actual strained lattice configurations, *i.e.* the BP approach. Therefore, more detailed calculations including the electronic (*e*^e^_*iν*_) and nuclear (*e*^n^_*iν*_) terms, and the total direct ‘proper’ (*e*_*iν*_) piezoelectric constants through the CPHF/KS approach are reported in Table 7 for the *Cmc*2_1_-X_2_PbO_3_ compounds. Evidently, each compound’s total direct ‘proper’ piezoelectric response contribution from the *e*^e^_*iν*_ are negligibly small and also reduce as X moves from K → Cs. The degree of polarization reduced resulting from a decrease in electronegativity down the periodic table group, which suggests that the contribution of *e*^e^_*iν*_ has diminished. Consequently, *e*^n^_*iν*_ accounts for the majority of each material’s total direct piezoelectric responses. Interestingly, the total direct and total direct ‘proper’ piezoelectric constants obtained from BP and CPHF/KS schemes agreed well for X = K, and Rb with deviations |*e*_*iν*_| < 0.02 C m^−2^. However, in the case of X = Cs, a comparatively high piezoelectric constant appears for *e*_31_ and *e*_33_ when CPHF/KS is adopted. This discrepancy may arise from the inherently more robust nature of the CPHF/KS scheme in evaluating e^n^_*iν*_, as it explicitly accounts for the partitioning of the nuclear-relaxation contribution of the piezoelectric tensor into phonon-mode-resolved components, together with the enhanced ionic polarizability introduced by the larger Cs cation. In such cases, the Berry-phase formalism, which predominantly captures the macroscopic polarization response, tends to emphasize long-range ionic contributions, whereas the CPHF/KS approach, being more sensitive to local field effects, reflects short-range electronic–ionic interactions. Consequently, the disparity between the two methods becomes more pronounced in Cs_2_PbO_3_ than in its lighter alkali counterparts. Overall, the maximum piezoelectric constants attributed by each compound are higher than the α-quartz, a standard piezoelectric material, whose largest constant *e*_11_ is 0.15 C m^−2^ at room temperature and 0.07 C m^−2^ at 5 K.^80^ Also, compared to the analogous compounds such as Na_2_SiO_3_ and Na_2_GeO_3_, our results show better responses than for Na_2_SiO_3_ (*e*_32_ = 0.22 C m^−2^), however they are lower than for Na_2_GeO_3_ (*e*_33_ = 0.90 C m^−2^).^12,28^

The electromechanical (EM) coupling factors (*k*_*iν*_) calculated using eqn (26) are presented in Table 7.^81^ This determines the efficiency of a piezoelectric material *i.e.*, the factor with which the materials convert mechanical energy into electrical energy or *vice versa*.26
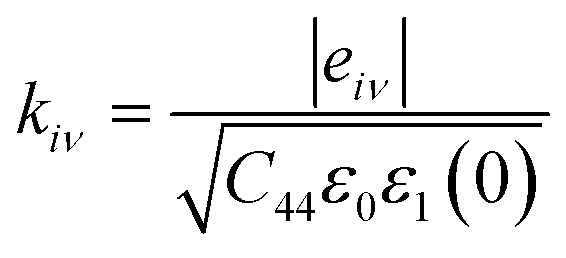
Here, *e*_*iν*_ are the piezoelectric constants, *C*_44_ is the elastic constant (in Pa unit), *ε*_0_ = 8.854 × 10^−12^ F m^−1^ is the absolute permittivity of free space and *ε*_1_(0) is the real static dielectric constant.

Firstly, it can be noticed that the maximum *k*_*iν*_ for each compound corresponds to the direction of their respective maximum piezoelectric constant. Even though the computed *k*_*iν*_ shows its highest value for X = Cs with *k*_33_ = 0.69, the overall coupling factors when all the directions are taken into account is highest for X = K, with X = Rb serving as an intermediary. This reveals a better efficiency of the electromechanical transducer between the stated electric and elastic channels for X = K, and Rb. Since our results of the maximum *k*_*iν*_ are higher than the quartz crystal^82^ with a highest electromechanical coupling coefficient of ∼0.29 in a direction which makes an angle of 73° with *y* axis in *yz* plane, the investigated *Cmc*2_1_-X_2_PbO_3_ materials could be potential candidates which can serve as efficient piezoelectric materials.

### 3.7 Thermoelectric properties

The primary focus of this work is to identify the TE efficiency (*ZT*) of the investigated compounds, which is closely related to the electron transport properties such as Seebeck coefficient (*S*), electrical conductivity (*σ*), electron thermal conductivity (*κ*_e_), and the lattice thermal conductivity (*κ*_L_). For this particular calculation, we have considered not only the p-type and n-type doping, but also the TE properties along the *x*, *y*, and *z*-axes by employing four different functionals such as PBE-GGA, B3LYP, HSE06, and PBE0. Herein, calculations for the electron transport properties are performed using the Boltzmann transport equation (BTE) within the CRTA at fixed *τ* = 10 fs (10^−14^ s) using BoltzTraP.^7^ The employed formulae for thermoelectric parameter calculations are:^83,84^27
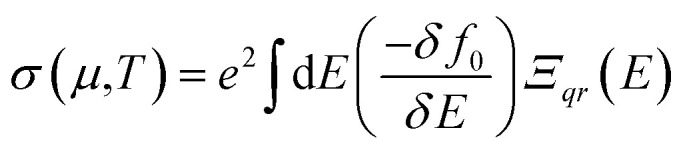
28
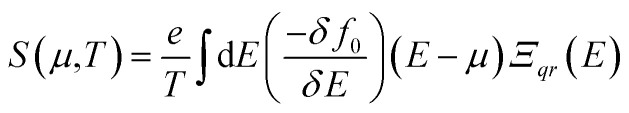
29
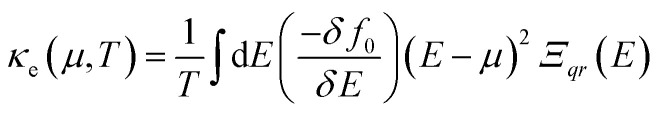
where, *µ* is the chemical potential or the Fermi level, *E* is the energy, *f*_0_ is the Fermi–Dirac distribution and *Ξ* is the kernel of the transport distribution function (TDF). Here, *Ξ* is further expressed as:30
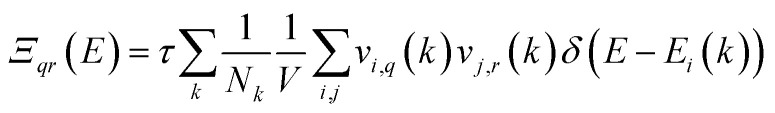
where, *v*_*i*,*q*_(*k*) is the velocity of the *i*^th^ band calculated along the Cartesian direction *q*, *τ* is the lifetime which is assumed to be constant according to the RTA. In the above equations, *σ* is the electrical conductivity, *S* is the Seebeck coefficient, and *κ*_e_ is the electronic thermal conductivity.

Here, the purpose for calculating the electronic transport properties is to obtain TE efficiency (figure of merit) *ZT*, given by31
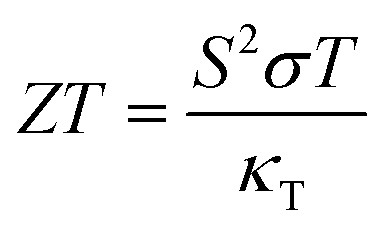
where, *κ*_T_ is the total thermal conductivity and is equal to *κ*_e_ + *κ*_L_. To determine the lattice contribution to the thermal conductivity (*κ*_L_), we employed the well-known Slack equation given by^85^32
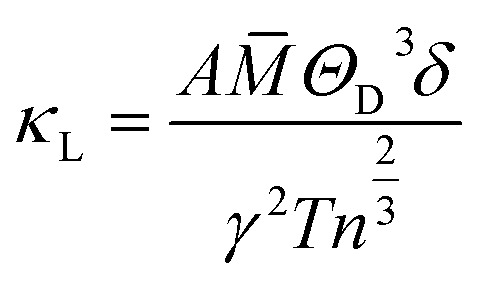
where, a constant 
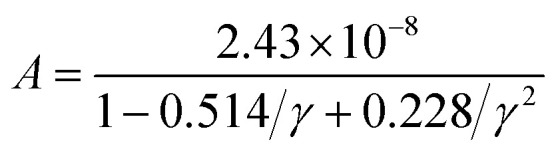
, *M̄* is average atomic mass, *Θ*_D_ is the Debye temperature, *δ*^3^ is the volume per atom, *γ* is the Gruneisen parameter, and *n* is the number of atoms per unit cell.

From the transport properties of electrons with respect to chemical potential (*µ*) presented in Fig. S2–S14, considering the direction-wise analysis, one can find that each of the transport properties for the investigated materials are highly anisotropic in nature with the *z*-axis the most preferable direction for achieving maximum TE efficiency except for in LPO where it is obtained along *x*-axis. Also, from these plots, it can be deduced that all of the compounds under investigation exhibit a p-type semi-conducting nature, with holes serving as the majority of carriers, regardless of the functionals employed. With hybrid functionals, each compound’s *E*_g_ increases which in turn raises the (*E* − *µ*) term of eqn (28). This subsequently maximizes the Seebeck coefficient (*S*) computed using hybrid-DFT when compared to the PBE-GGA results [see Fig. S9–S11]. Also, on analyzing and comparing the PBE-GGA and hybrid-DFT band structure profiles of the investigated compounds [see Fig. 5 and S1], it is evident that the incorporated hybrid functionals have had a notable impact, particularly with regard to the energy levels where the electronic band lines are positioned. Thus, these substantial variations in band profiles due to the functionals employed has led to considerable changes in the ensuing transport properties. The resultant change in band energy due to the different functionals being adopted has led to the change in density of states, being a key factor for which the term −*δf*_0_/*δE* of eqn (27)–(29), that defines the number of states mapped and offered information of the channel allowing the charges to flow, varies. This phenomenon leads to remarkable changes of each compound’s electrical conductivity (*σ*) and electron thermal conductivity (*κ*_e_), when the PBE-GGA and hybrid-DFT computed transport properties are compared.

**Fig. 9:**
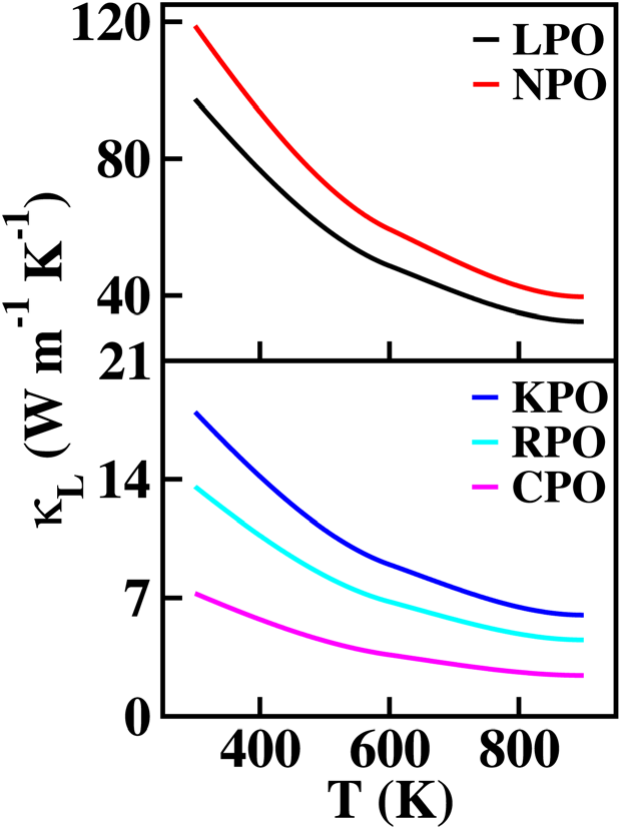
Calculated lattice thermal conductivity (*κ*_L_) of X_2_PbO_3_ compounds using the Slack equation.

**Fig. 10:**
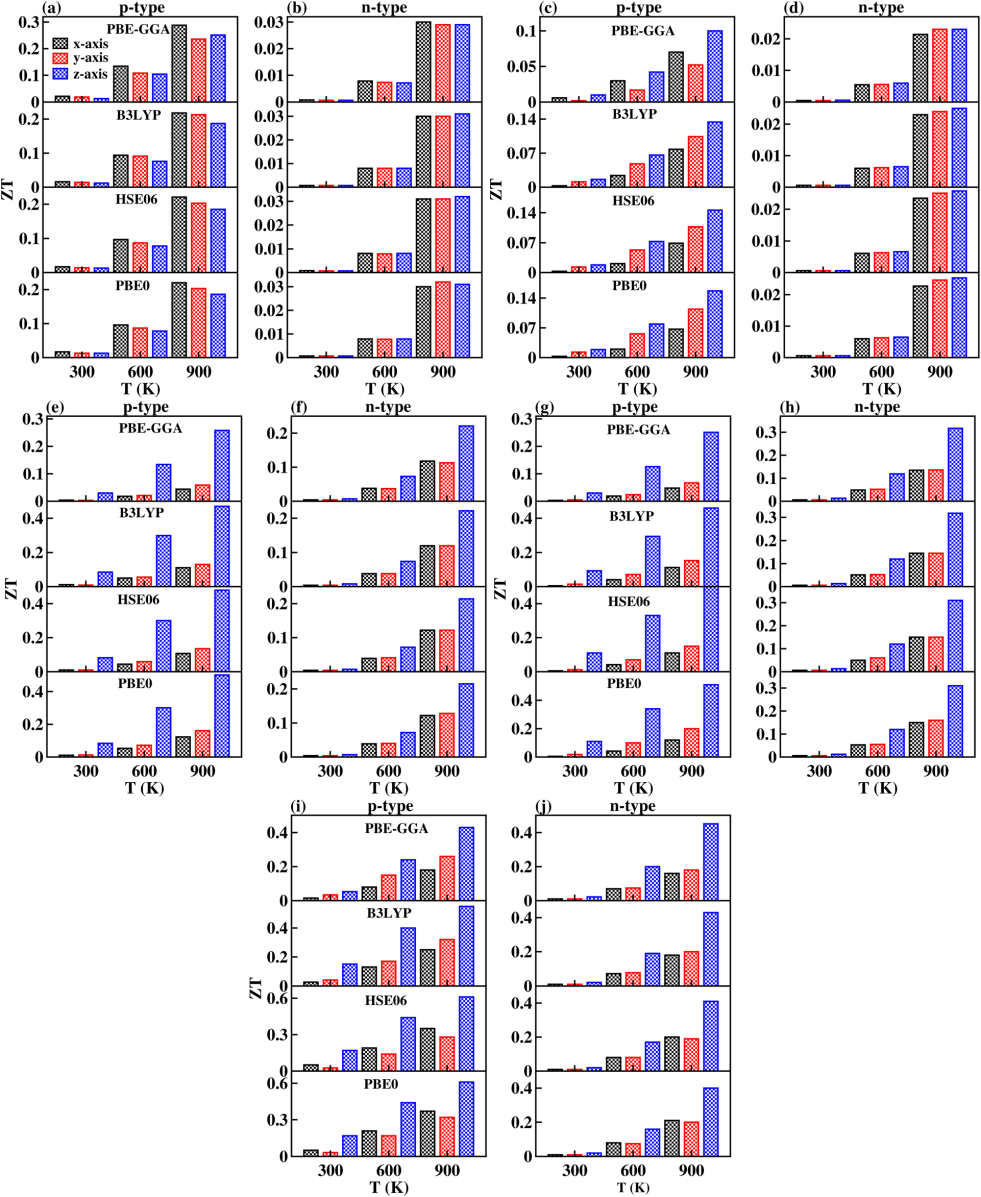
Calculated thermoelectric efficiency (*ZT*) as a function of temperature using PBE-GGA, B3LYP, HSE06, and PBE0 functionals: (a) p-type and (b) n-type for Li_2_PbO_3_, (c) p-type and (d) n-type for Na_2_PbO_3_, (e) p-type and (f) n-type for K_2_PbO_3_, (g) p-type and (h) n-type for Rb_2_PbO_3_, and (i) p-type and (j) n-type for Cs_2_PbO_3_.

The primary aim for calculating transport properties is to obtain the figure of merit (*ZT*) which determines the material’s TE efficiency. In this regard, the TE power factor (PF = *S*^2^*σ*) is firstly computed that measures the TE performance of the materials. The PFs with respect to chemical potential plots are depicted in Fig. S12–S14. From these plots, it is revealed that the PF increased as temperature rises, suggesting the enhancement of TE efficiency of the materials with temperature. It is important to note that the first peak points of PF along the left and right sides of the zero chemical potential (Fermi energy) corresponds to the optimum chemical potentials for p-type and n-type doping at various temperatures, where the optimal *ZT* values are determined as a function of temperature. Obviously, the *ZT* [see eqn (31)] depends on the conflicting nature of *σ* and *κ*_e_, *S* and also on the *κ*_L_. Since, the transport properties from BoltzTraP does not generate *κ*_L_, therefore an analytical Slack model given in eqn (32) is employed which shows reliable *κ*_L_ values for complex materials compared to *κ*_L_ estimated from the phonon BTE calculations.^86^ The obtained *κ*_L_ curves in Fig. 9 show continuously decreasing plots as the temperature rises due to the increasing phonon scattering. The larger atomic sizes as X goes from K → Cs for *Cmc*2_1_-X_2_PbO_3_ materials result in a reduced possibility of phonon scattering down the group. Thus, the order of *κ*_L_ for *Cmc*2_1_-X_2_PbO_3_ materials is KPO > RPO > CPO. However, our observation shows a conflicting nature for *C*2/*c*-X_2_PbO_3_ compounds where *κ*_L_ of NPO > *κ*_L_ of LPO. This might be due to the LPO attaining a higher density than NPO, which decreases heat transfer average distances, and therefore minimizes *κ*_L_ for LPO. Furthermore, the non-centrosymmetric distortions in the *Cmc*2_1_-X_2_PbO_3_ phase contribute to enhanced phonon scattering, leading to lower *κ*_L_ values and improved thermoelectric performance relative to the centrosymmetric *C*2/*c*-X_2_PbO_3_ counterparts. Additionally, the calculated *κ*_L_ values for *Cmc*2_1_-X_2_PbO_3_ compounds are lower than those of the structurally related *Cmc*2_1_-Li_2_GeO_3_ compounds, which can be attributed to the presence of the heavy Pb atom.^87^ The increased atomic mass of Pb further suppresses phonon transport, reinforcing the observed reduction in *κ*_L_. Evidently, from the *ZT* plot as a function of temperature shown in Fig. 10, it can be seen that each compound’s *ZT* value improves as the temperature increases. Furthermore, it can be determined that CPO has attained the best TE efficiency with *ZT* = 0.61 at *T* = 900 K along the *z*-direction for p-type doping with the PBE0 functional. Interestingly, each material’s computed *ZT* from hybrid-DFT shows better efficiency when compared to the PBE-GGA result, except for LPO where the PBE-GGA estimates the greatest *ZT* values. The present findings are noteworthy, even though the achieved TE efficiency remains moderate (given that the benchmark for high-performance thermoelectric materials is *ZT* ≥ 1). This study represents the first theoretical report on the TE properties of *C*2/*c*-X_2_PbO_3_ and *Cmc*2_1_-X_2_PbO_3_ glass-like compounds, which exhibit appreciable *ZT* values at elevated temperatures. Nevertheless, further in-depth investigations are required to enhance the electrical conductivity, particularly for the *Cmc*2_1_-X_2_PbO_3_ phase, to realize their potential applicability in future thermoelectric devices. In general, glass-like materials crystallizing in the orthorhombic *Cmc*2_1_ phase are known to possess relatively low electrical conductivity, which intrinsically restricts their TE efficiency. Consequently, only limited studies have explored this class of compounds. Reported data indicate that pristine glass-like Li_2_GeO_3_ displays a maximum *ZT* of merely 0.35 at 1200 K, which is significantly lower than the values obtained in the present work, underscoring the comparative promise of the investigated systems.^87^ This finding suggests that, within the family of glass-like materials, X_2_PbO_3_ holds considerable promise as a next-generation thermoelectric candidate.

## 4 Conclusion

We present a comprehensive investigation of the multi-functional properties of X_2_PbO_3_ (X = Li, Na, K, Rb, and Cs) using hybrid-DFT within the frameworks of B3LYP, HSE06, and PBE0 functionals. Our study reveals that X_2_PbO_3_ compounds crystallize in two distinct structural phases, namely the monoclinic (*C*2/*c*) and orthorhombic (*Cmc*2_1_) symmetries, depending on the ionic radius of the X-site atoms. To ensure the practical feasibility of these materials, we conducted a series of stability assessments: (1) the room temperature stability (from MD-simulation), (2) mechanical stability confirmed using Born’s criteria, and (3) ground-state stability or energetic stability established from formation energy calculations. The electronic band structure analysis demonstrates a significant band gap increasing within the hybrid functionals, highlighting the semiconducting nature of X_2_PbO_3_. From the optical property calculations, we observed a strong absorption coefficient (*α* > 1 × 10^5^ cm^−1^) within the ultraviolet (UV) region, suggesting their potential use as UV-radiation shielding materials. The piezoelectric properties of the *Cmc*2_1_-X_2_PbO_3_ phase were evaluated using both the PB and CPHF/KS formalism. The calculated piezoelectric coefficients are comparable to those of benchmark piezoelectric materials such as α-quartz (α-SiO_2_), underscoring the promising electromechanical potential of X_2_PbO_3_. Nevertheless, the effective macroscopic performance in practical devices will depend on factors including crystallographic orientation, poling conditions, and domain engineering, which can modulate the intrinsic response predicted here. Complementary thermoelectric analyses further reveal that these compounds exhibit favorable high-temperature transport characteristics, indicating their suitability for waste-heat recovery and energy-conversion applications. Overall, this work demonstrates the multifunctional potential of X_2_PbO_3_ for piezoelectric, thermoelectric, and optoelectronic applications. Experimental validation is encouraged to substantiate the predicted responses. Moreover, the present findings offer valuable guidance for computational researchers in selecting hybrid functionals optimized for reproducing structural parameters in DFT-based studies. Future efforts incorporating intrinsic defects, vacancies, or intentional doping are expected to enhance the thermoelectric performance and advance these materials toward practical device-level implementation.

## Author contributions

R. Zosiamliana: performed detail calculations, formal analysis, visualization, validation, literature review, writing – original draft, writing – review & editing. Lalhriat Zuala: formal analysis, visualization, validation, writing – review & editing. Lalrinthara Pachuau: formal analysis, visualization, validation, writing – review & editing. Lalmuanpuia Vanchhawng: formal analysis, visualization, validation, writing – review & editing. S. Gurung: formal analysis, visualization, validation, writing – review & editing. A. Laref: formal analysis, visualisation, validation, writing – review & editing. Shalika Ram Bhandari: formal analysis, visualisation, validation, writing – review & editing. D. P. Rai: project management, supervision, resources, software, formal analysis, visualisation, validation, writing – review & editing.

## Conflicts of interest

The authors declare no competing financial interest

## Supplementary Material

RA-015-D5RA07886E-s001

## Data Availability

All data supporting the findings of this study are available within the article and its supplementary information (SI). Additional datasets generated and/or analysed during the current study are available from the corresponding author on reasonable request. Supplementary information is available. See DOI: https://doi.org/10.1039/d5ra07886e.
