# Supplementary material for: Group-I lead oxide X2PbO3 (X = Li, Na, K, Rb, and Cs) glass-like materials for energy applications: a hybrid-DFT study
Source: RSC Adv. 2025 Dec 18;15(59):50881–97. doi: 10.1039/d5ra07886e (PMC12713738; doi:10.1039/d5ra07886e)
Supplement: RA-015-D5RA07886E-s001 [file RA-015-D5RA07886E-s001.pdf]

Supplementary Information (SI) for RSC Advances.
This journal is © The Royal Society of Chemistry 2025

Supplementary data

Group-I lead oxide X_2PbO_3 (X=Li, Na, K, Rb, and Cs) glass-like materials for energy applications: A hybrid-DFT study

R. Zosiamliana,[a,b] Lalhriat Zuala,[b] Lalrinthara Pachuau, [b] Lalmuanpuia Vanchhawng, [b] S. Gurung,[b] A. Laref, [c] Shalik R. Bhandari,[d] and D. P. Rai [a,*]

[a] Department of Physics, Mizoram University, Aizawl-796004, India.
[b] Physical Sciences Research Center (PSRC), Department of Physics, Pachhunga University College, Aizawl-796001, India.
[c] Department of Physics and Astronomy, College of Science, King Saud University, Riyadh, 11451, Saudi Arabia.
[d] Department of Physics, Bhairahawa Multiple Campus, Tribhuvan University, Siddarthanagar-32900, Rupandehi, Nepal.

*__Email:__ *dibyaprakashrai@gmail.com*

Table S1: Calculated structural parameters and formation energy (E^f) for X_2PbO_3 (X=Li, Na, K, Rb, Cs) compounds. Here, |Δa|, |Δb|, and |Δc| represent the relative error percentages of lattice parameters with respect to the experimental data. Also, * is the suited functional in reproducing the experimental lattice parameters more accurately.

Symmetry (Space group)	Employed Methodology	a (Å)	b (Å)	c (Å)	α	β	γ	|a| (%)	|b| (%)	|c| (%)	E^f (Ha)
X=Li											
C2/c	PBE-GGA	5.50	9.49	5.57	90.00°	110.44°	90.00°	0.92	2.48	1.64	-0.126
	B3LYP	5.52	9.39	5.58	90.00°	111.42°	90.00°	0.73	1.40	1.82	-0.159
	HSE06	5.43	9.31	5.49	90.00°	110.70°	90.00°	0.37	0.54	0.18	-0.159
	PBE0*	5.42	9.30	5.48	90.00°	110.68°	90.00°	0.55	0.43	0.00	-0.166
	Exp. [1]	5.45	9.26	5.48	90.00°	111.22°	90.00°	---	---	---	---
X=Na											
C2/c	PBE-GGA	6.01	10.03	5.79	90.00°	111.42°	90.00°				-0.123
	B3LYP	5.98	9.94	5.75	90.00°	111.57°	90.00°				-0.154
	HSE06	5.93	9.85	5.68	90.00°	111.45°	90.00°				-0.155
	PBE0	5.92	9.83	5.67	90.00°	111.46°	90.00°				-0.162
	Exp.	---	---	---	---	---	---	---	---	---	---
X=K											
C*mc*2_1	PBE-GGA	10.93	7.09	6.13	90.00°	90.00°	90.00°	2.73	0.00	2.51	-0.140
	B3LYP	10.88	7.06	6.10	90.00°	90.00°	90.00°	2.26	0.42	2.01	-0.170
	HSE06	10.78	7.03	6.03	90.00°	90.00°	90.00°	1.32	0.85	0.84	-0.173
	PBE0*	10.77	7.03	6.02	90.00°	90.00°	90.00°	1.22	0.85	0.67	-0.179
	Exp. [2]	10.64	7.09	5.98	90.00°	90.00°	90.00°	---	---	---	---
X=Rb											
C*mc*2_1	PBE-GGA	11.16	7.51	6.16	90.00°	90.00°	90.00°	2.95	0.27	2.50	-0.166
	B3LYP	11.12	7.49	6.13	90.00°	90.00°	90.00°	2.58	0.00	1.99	-0.197
	HSE06	10.99	7.44	6.04	90.00°	90.00°	90.00°	1.38	0.67	0.50	-0.202
	PBE0*	10.97	7.44	6.03	90.00°	90.00°	90.00°	1.20	0.67	0.33	-0.210
	Exp. [3]	10.84	7.49	6.01	90.00°	90.00°	90.00°	---	---	---	---
X=Cs											
C*mc*2_1	PBE-GGA	11.62	7.96	6.22	90.00°	90.00°	90.00°	2.65	1.53	2.81	-0.172
	B3LYP	11.64	8.01	6.20	90.00°	90.00°	90.00°	2.83	2.17	2.48	-0.204
	HSE06	11.47	7.89	6.11	90.00°	90.00°	90.00°	1.33	0.64	0.99	-0.207

	PBE0*	11.46	7.89	6.09	90.00^{O}	90.00^{O}	90.00^{O}	1.24	0.64	0.66	-0.215
	Exp. [5]	11.32	7.84	6.05	90.00^{O}	90.00^{O}	90.00^{O}	---	---	---	---

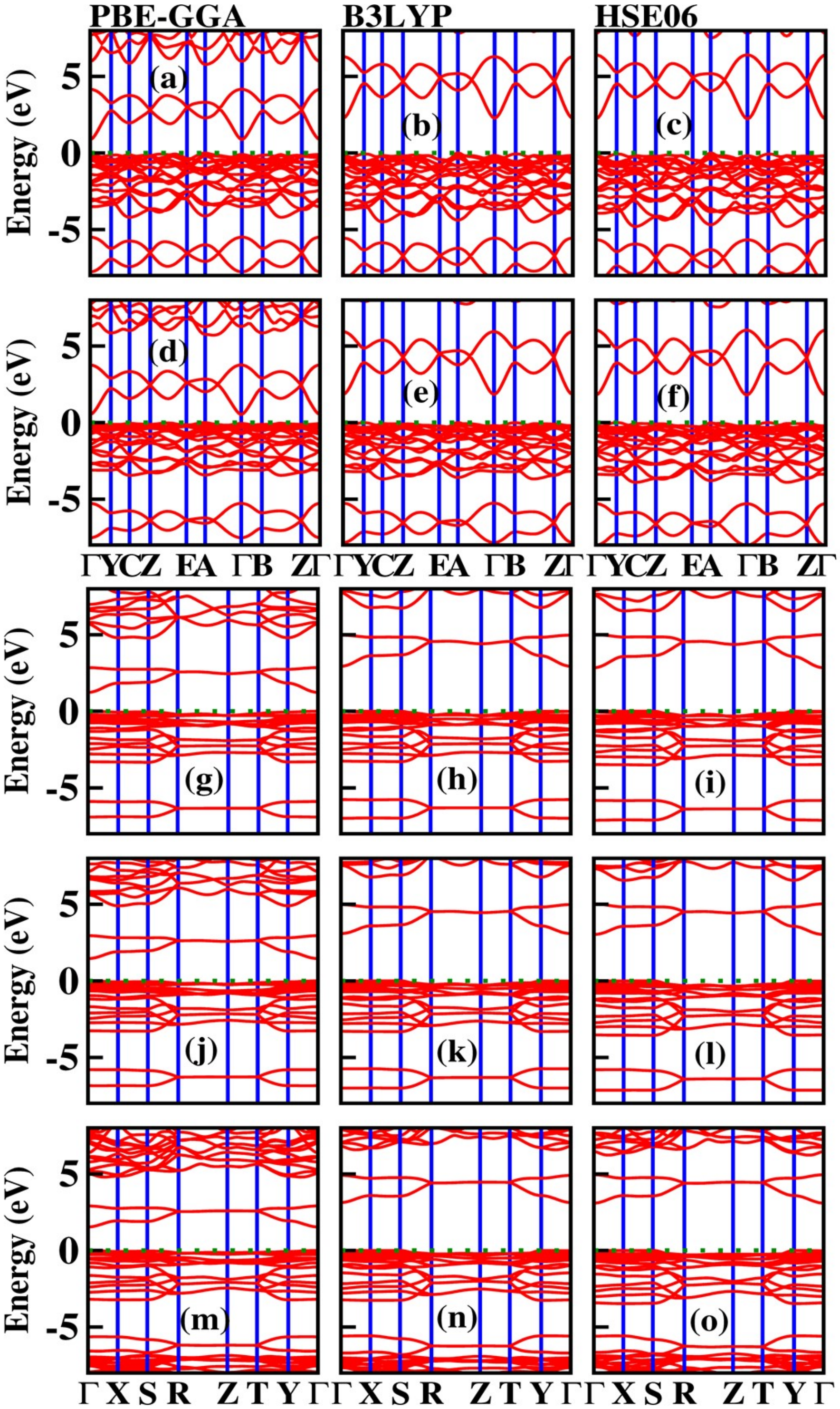

FIG. S1: Calculated band structures for X_2PbO_3 using PBE-GGA, B3LYP, and HSE06 and PBE0 functionals: (a-c) X=Li, (d-f) X=Na, (g-i) X=K, (j-l) X=Rb, and (m-o) X=Cs.

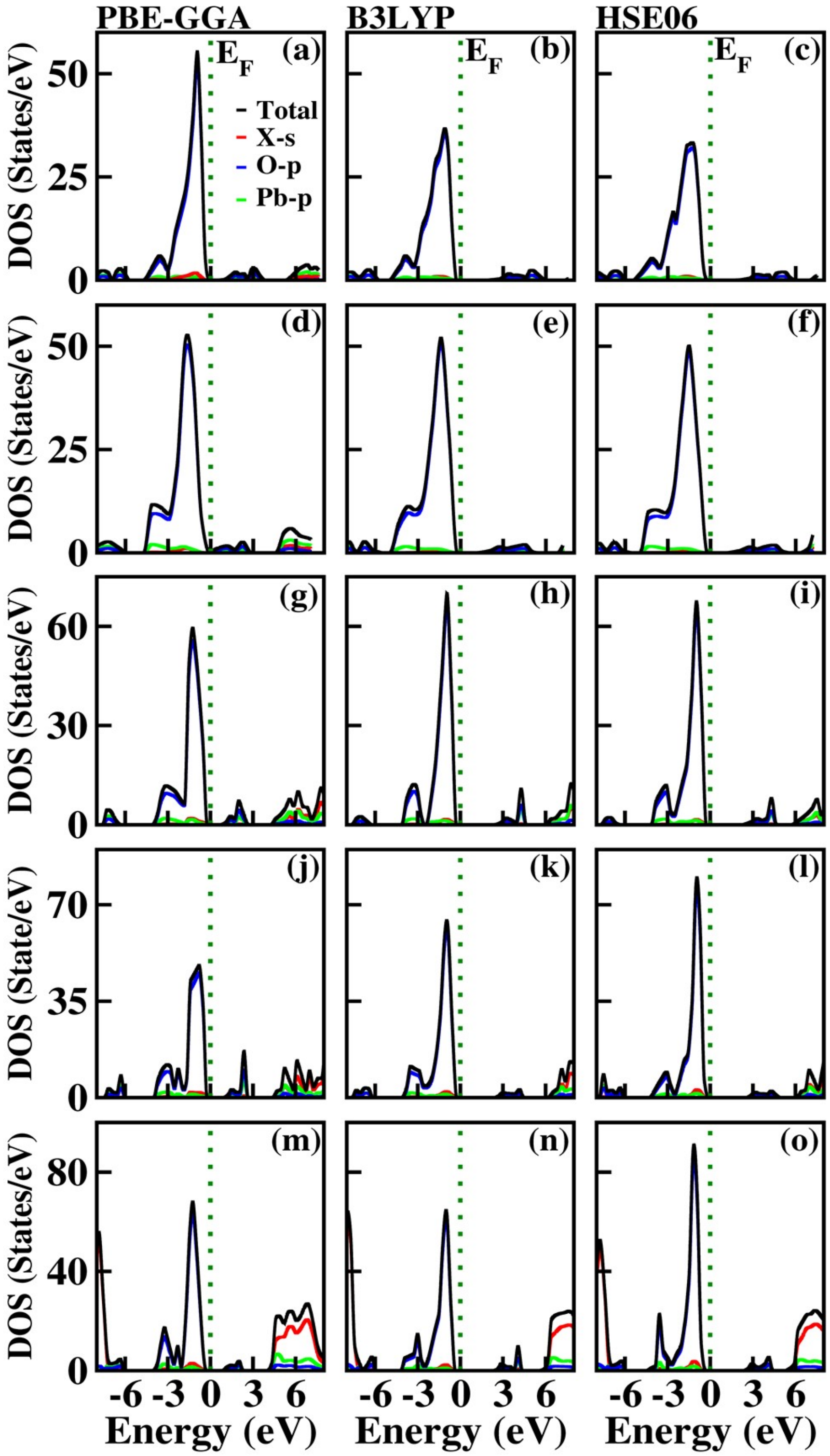

FIG. S2: Calculated DOS for X_2PbO_3 using PBE-GGA, B3LYP, and HSE06 and PBE0 functionals: (a-c) X=Li, (d-f) X=Na, (g-i) X=K, (j-l) X=Rb, and (m-o) X=Cs.

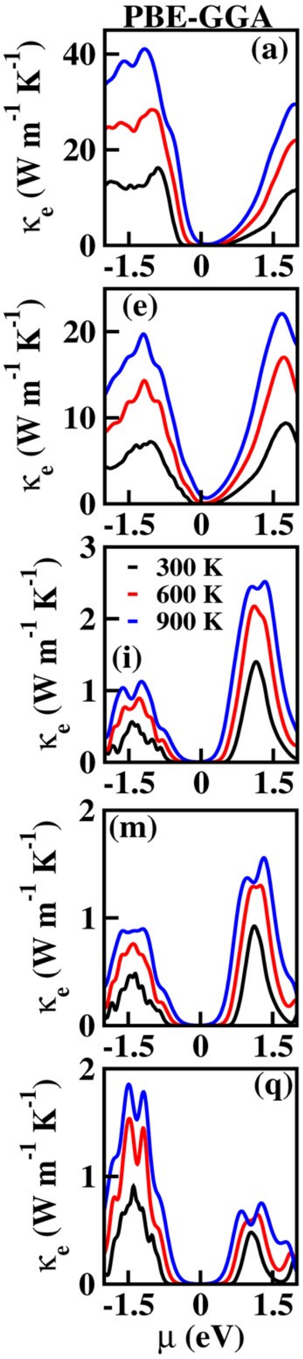

FIG. S3: Calculated electronic contribution to thermal conductivity (κ_e) for X_2PbO_3 along x-axis using PBE-GGA, B3LYP, HSE06, and PBE0 functionals: (a-d) X=Li, (e-h) X=Na, (i-l) X=K, (m-p) X=Rb, and (q-t) X=Cs.

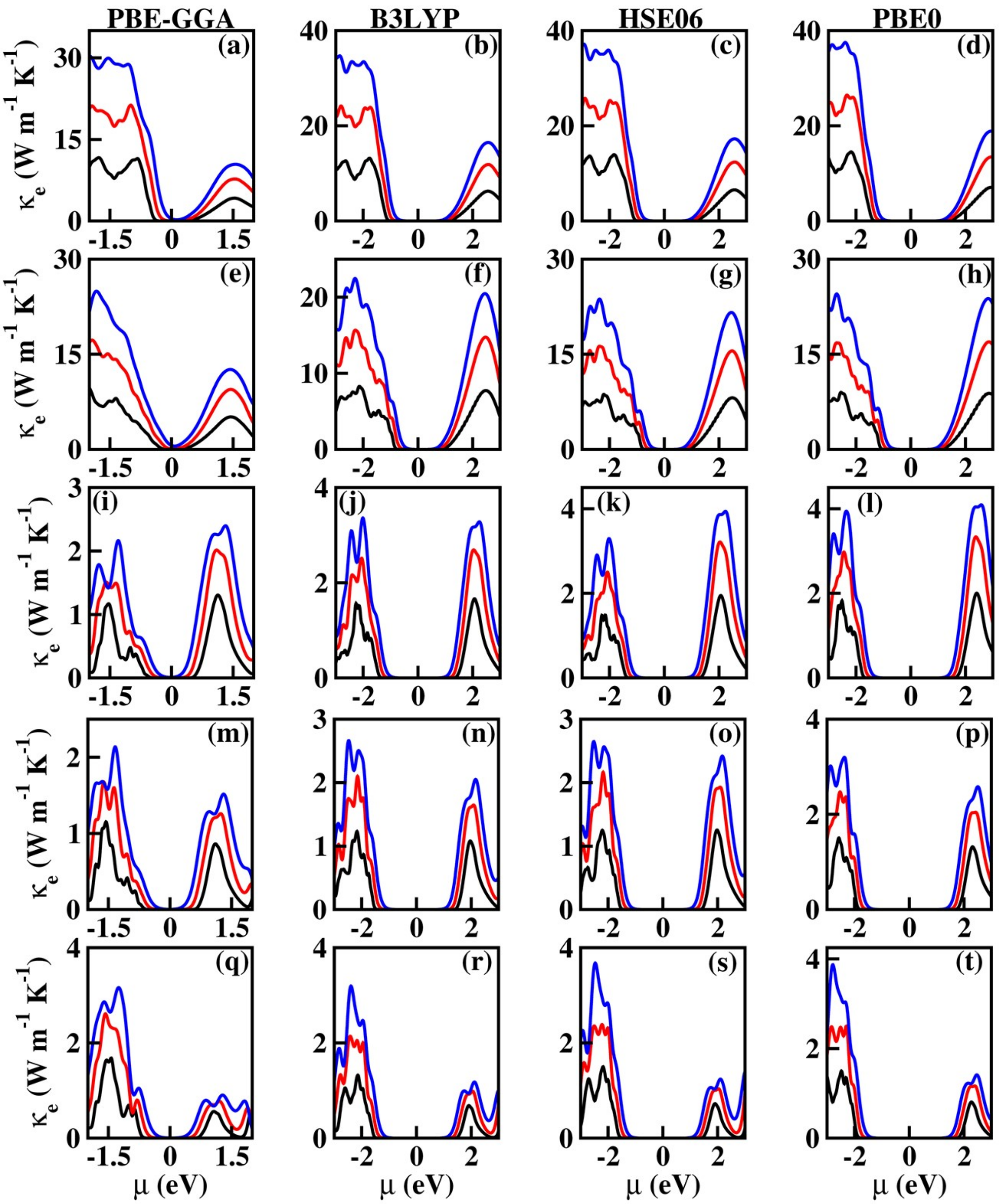

FIG. S4: Calculated electronic contribution to thermal conductivity (κ_e) for X_2PbO_3 along y-axis using PBE-GGA, B3LYP, HSE06, and PBE0 functionals: (a-d) X=Li, (e-h) X=Na, (i-l) X=K, (m-p) X=Rb, and (q-t) X=Cs.

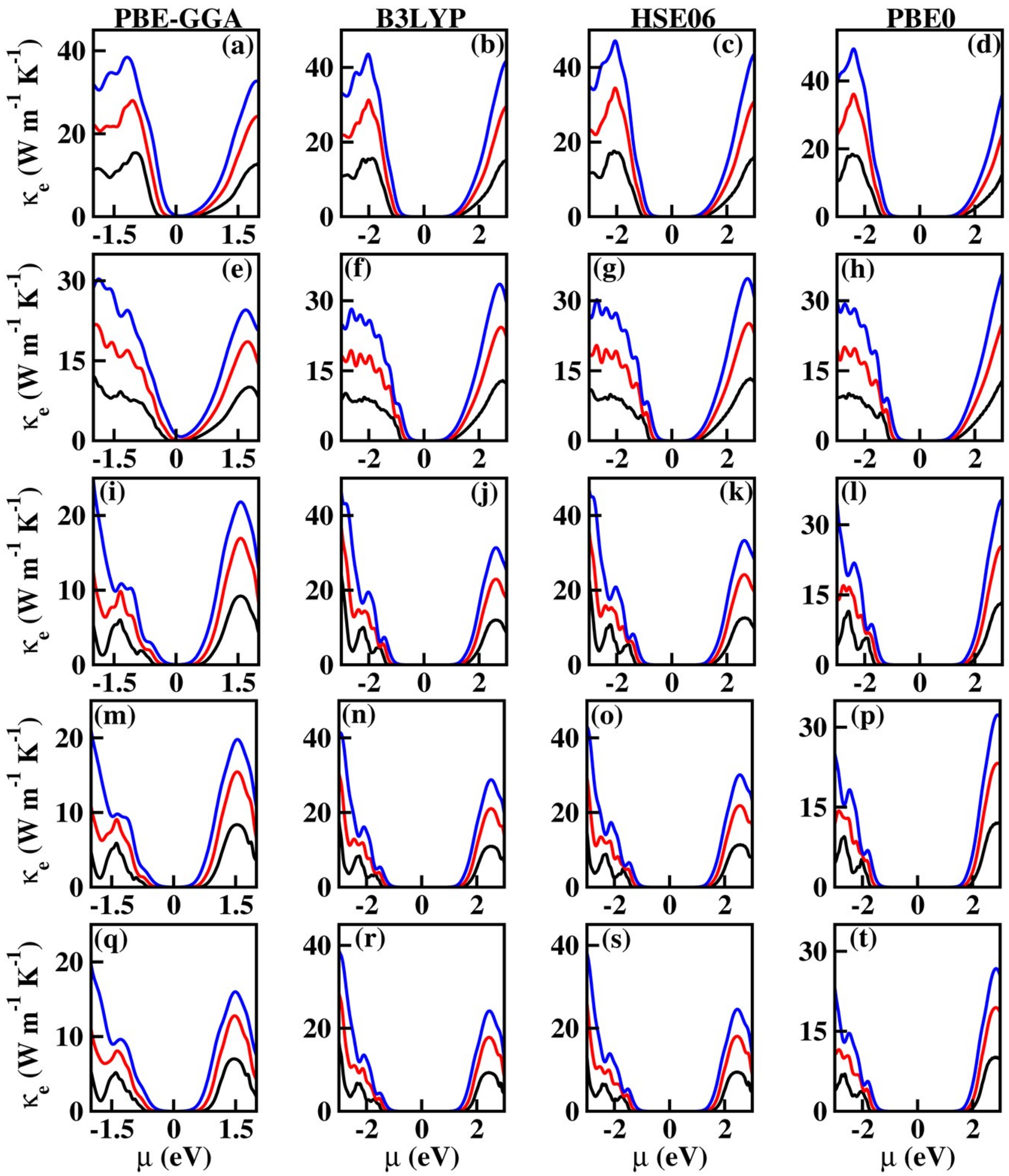

FIG. S5: Calculated electronic contribution to thermal conductivity (κ_e) for X_2PbO_3 along z-axis using PBE-GGA, B3LYP, HSE06, and PBE0 functionals: (a-d) X=Li, (e-h) X=Na, (i-l) X=K, (m-p) X=Rb, and (q-t) X=Cs.

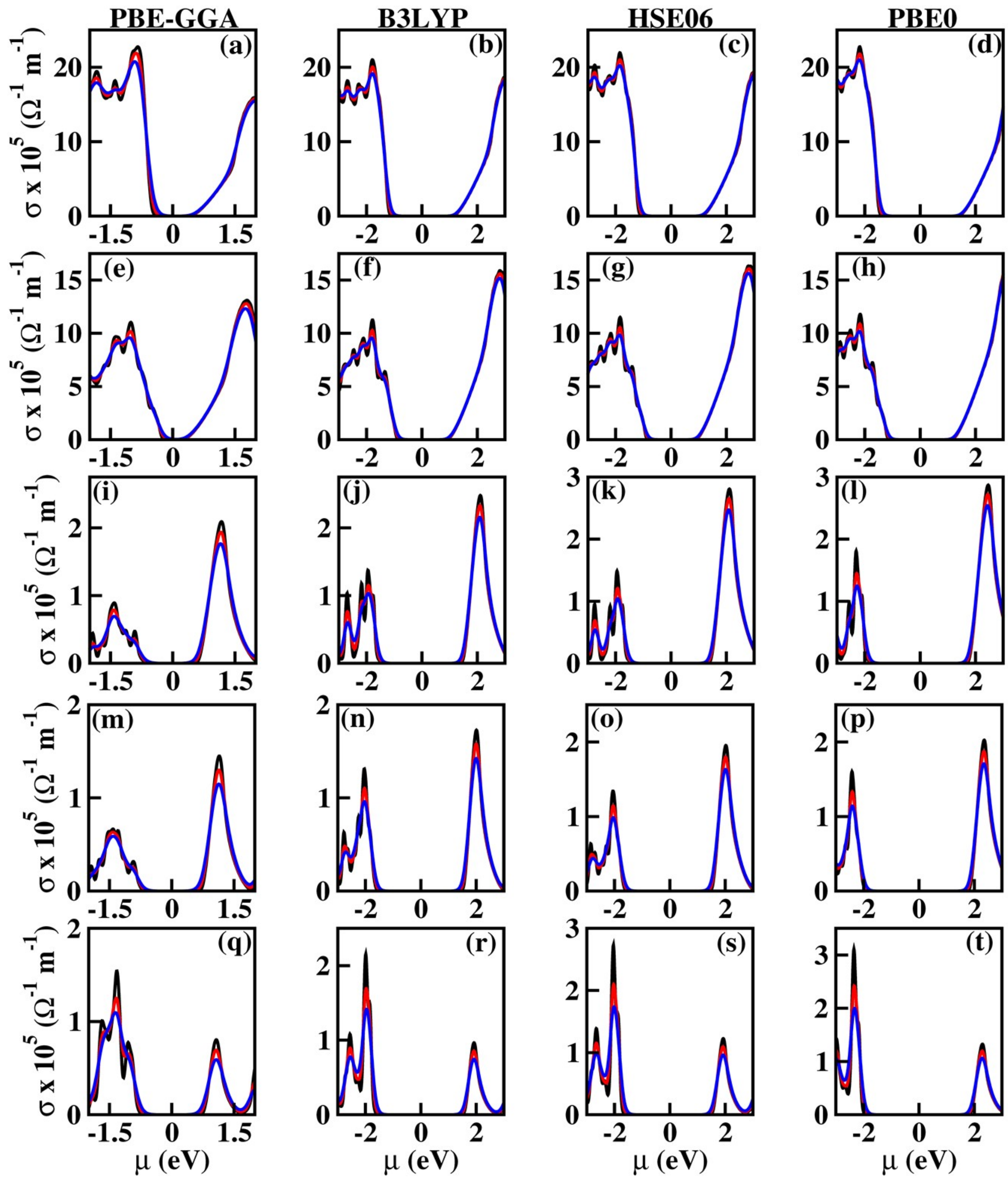

FIG. S6: Calculated electrical conductivity (σ) for X_2PbO_3 along x-axis using PBE-GGA, B3LYP, HSE06, and PBE0 functionals: (a-d) X=Li, (e-h) X=Na, (i-l) X=K, (m-p) X=Rb, and (q-t) X=Cs.

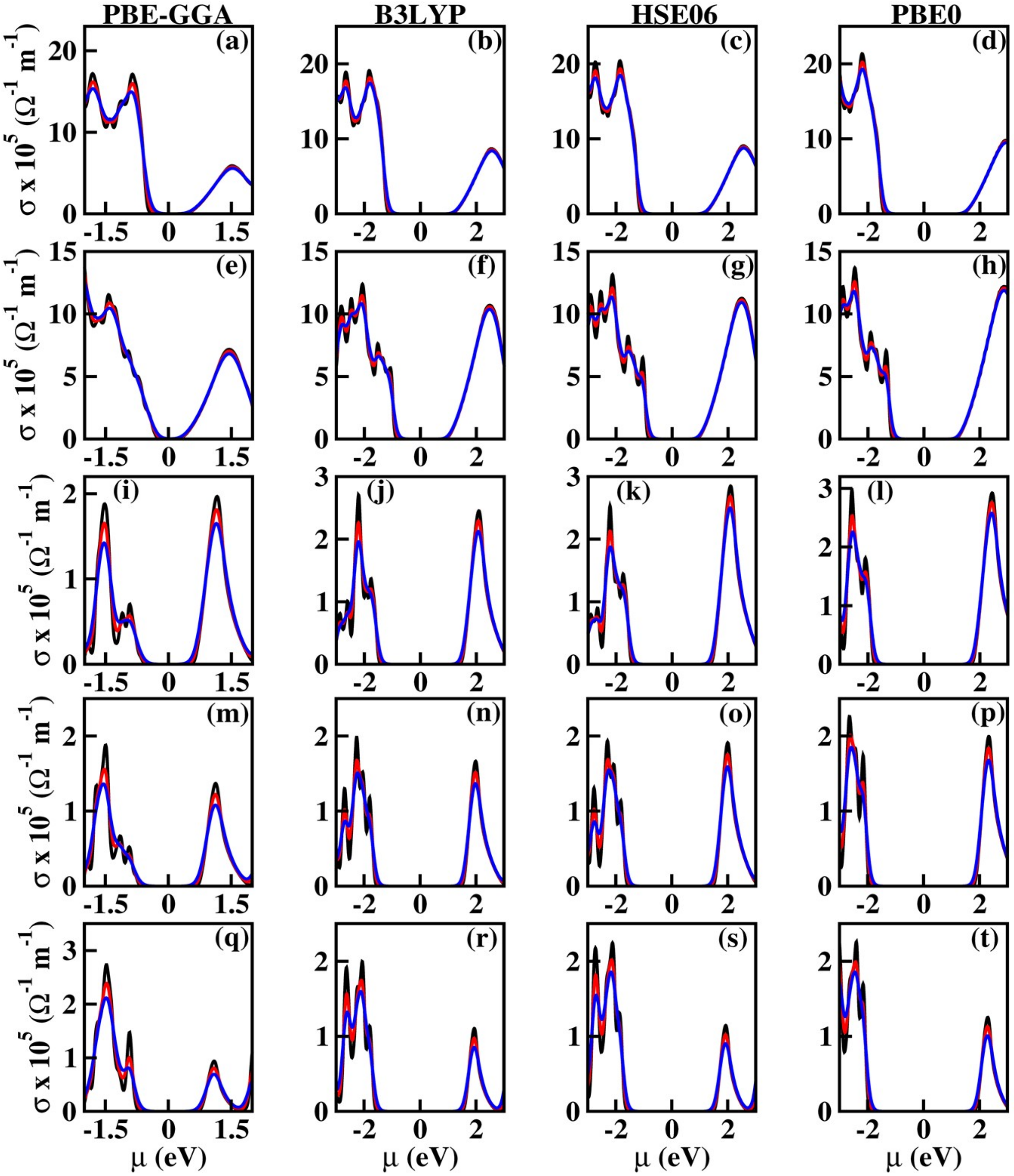

FIG. S7: Calculated electrical conductivity (σ) for X_2PbO_3 along y-axis using PBE-GGA, B3LYP, HSE06, and PBE0 functionals: (a-d) X=Li, (e-h) X=Na, (i-l) X=K, (m-p) X=Rb, and (q-t) X=Cs.

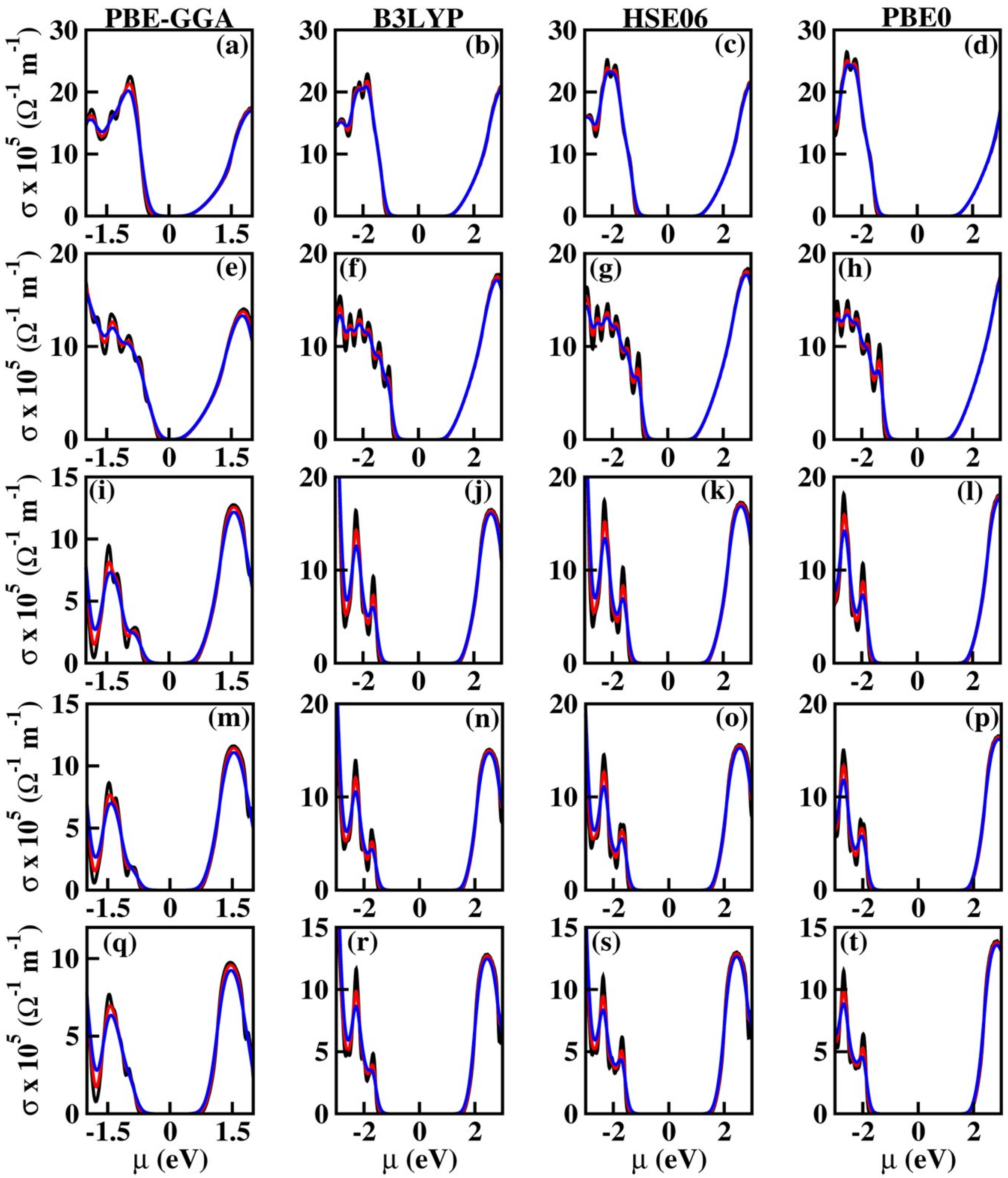

FIG. S8: Calculated electrical conductivity (σ) for X_2PbO_3 along z-axis using PBE-GGA, B3LYP, HSE06, and PBE0 functionals: (a-d) X=Li, (e-h) X=Na, (i-l) X=K, (m-p) X=Rb, and (q-t) X=Cs.

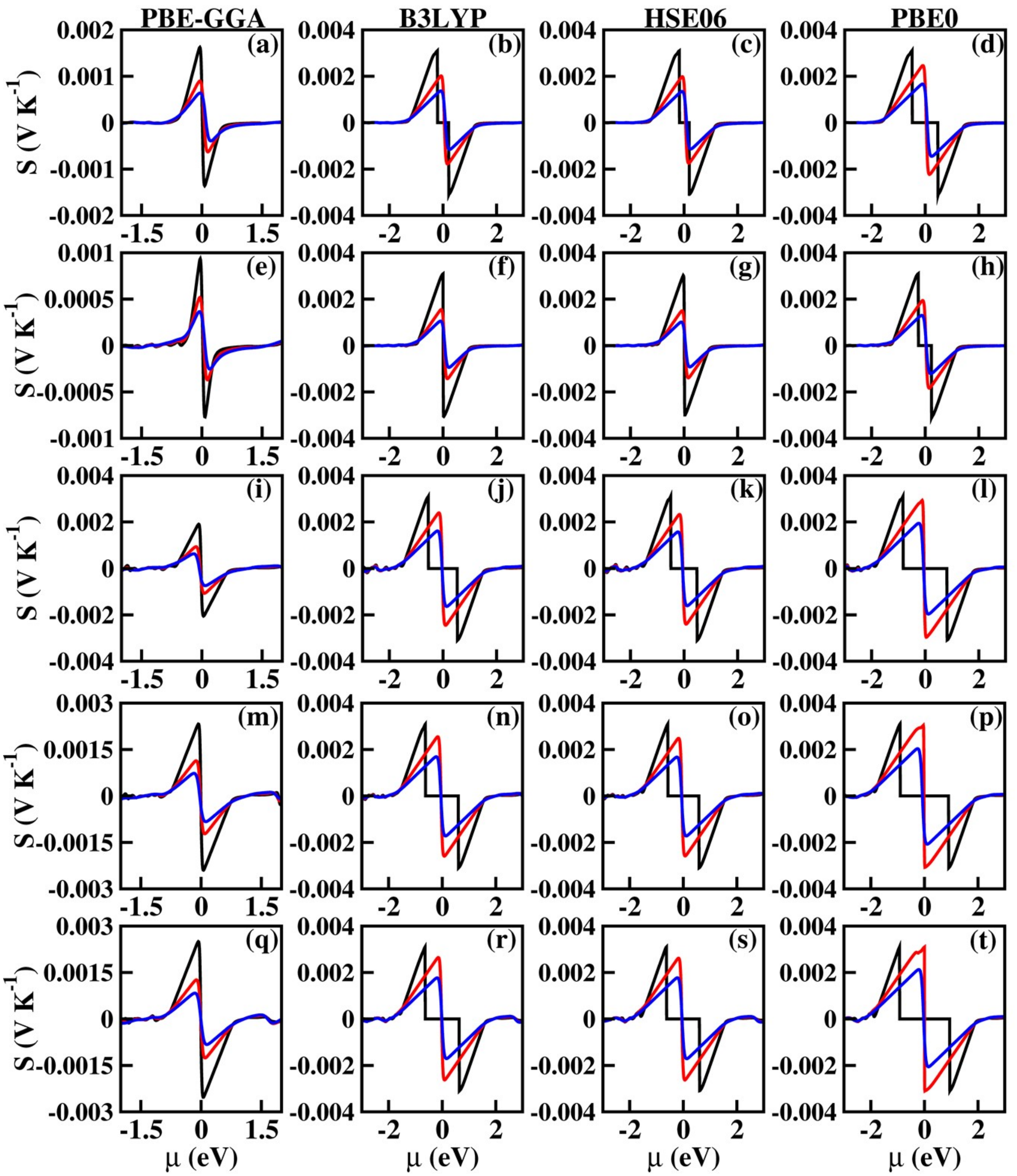

FIG. S9: Calculated Seebeck coefficient (S) for X_2PbO_3 along x-axis using PBE-GGA, B3LYP, HSE06, and PBE0 functionals: (a-d) X=Li, (e-h) X=Na, (i-l) X=K, (m-p) X=Rb, and (q-t) X=Cs.

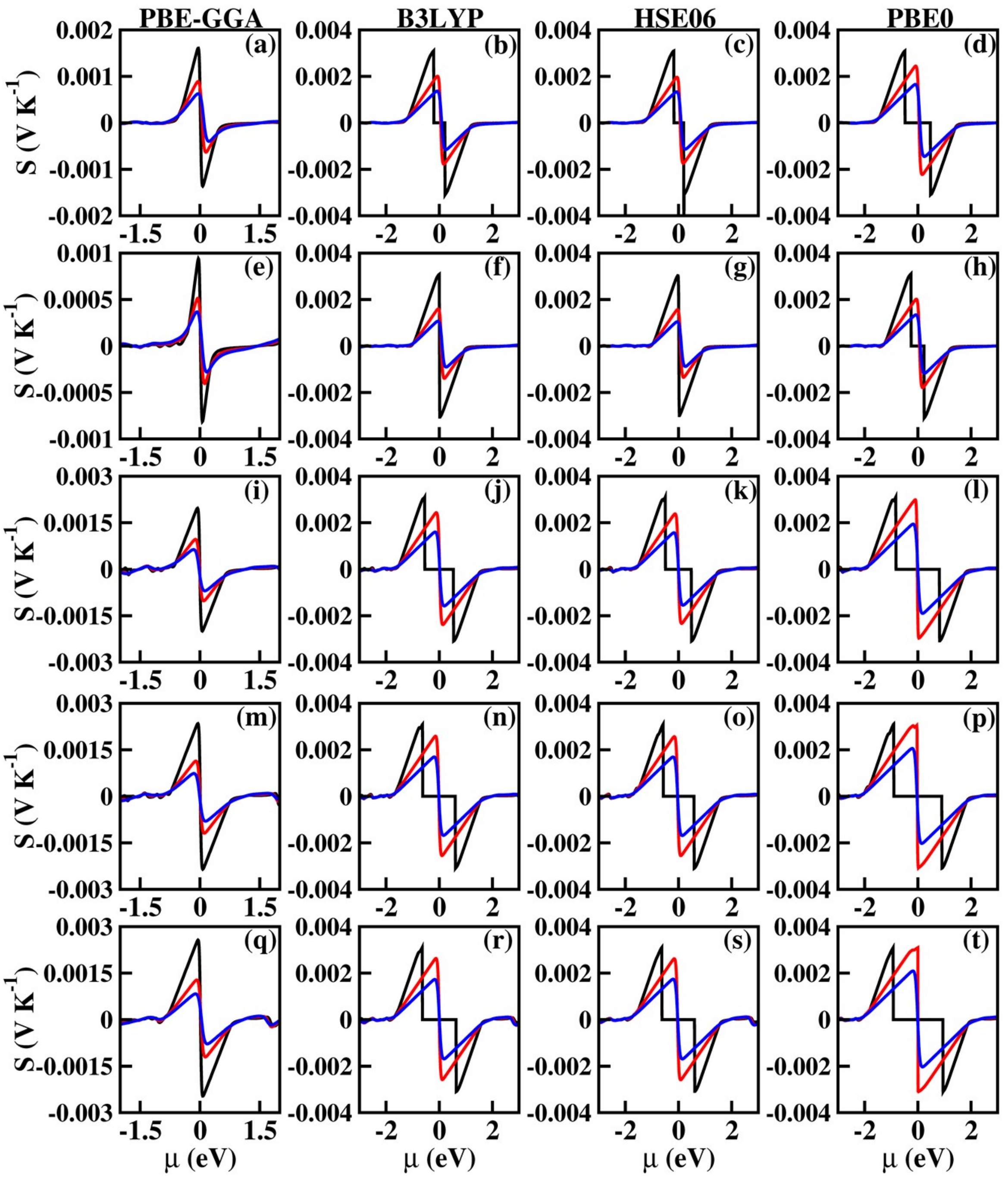

FIG. S10: Calculated Seebeck coefficient (S) for X_2PbO_3 along y-axis using PBE-GGA, B3LYP, HSE06, and PBE0 functionals: (a-d) X=Li, (e-h) X=Na, (i-l) X=K, (m-p) X=Rb, and (q-t) X=Cs.

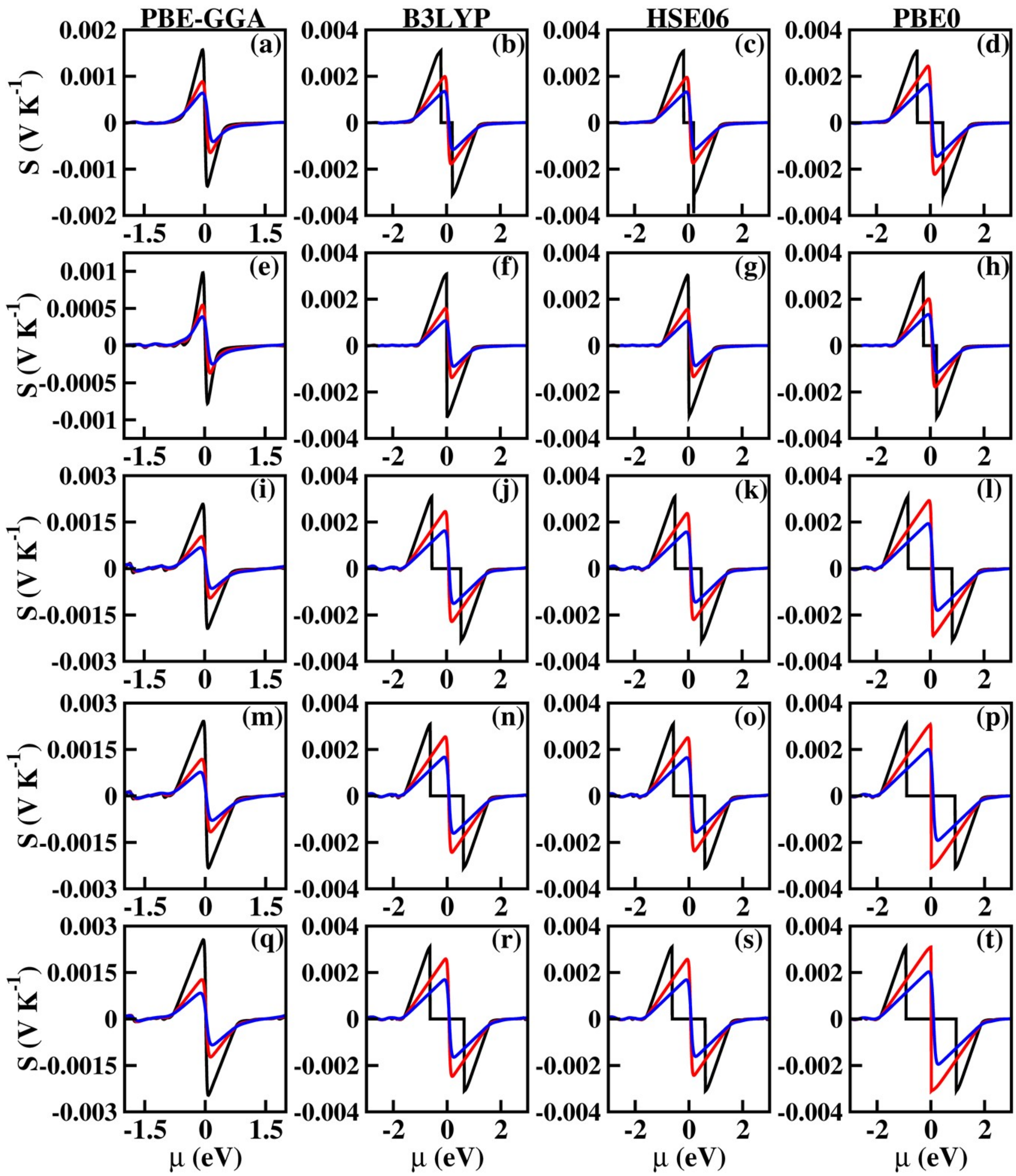

FIG. S11: Calculated Seebeck coefficient (S) for X_2PbO_3 along z-axis using PBE-GGA, B3LYP, HSE06, and PBE0 functionals: (a-d) X=Li, (e-h) X=Na, (i-l) X=K, (m-p) X=Rb, and (q-t) X=Cs.

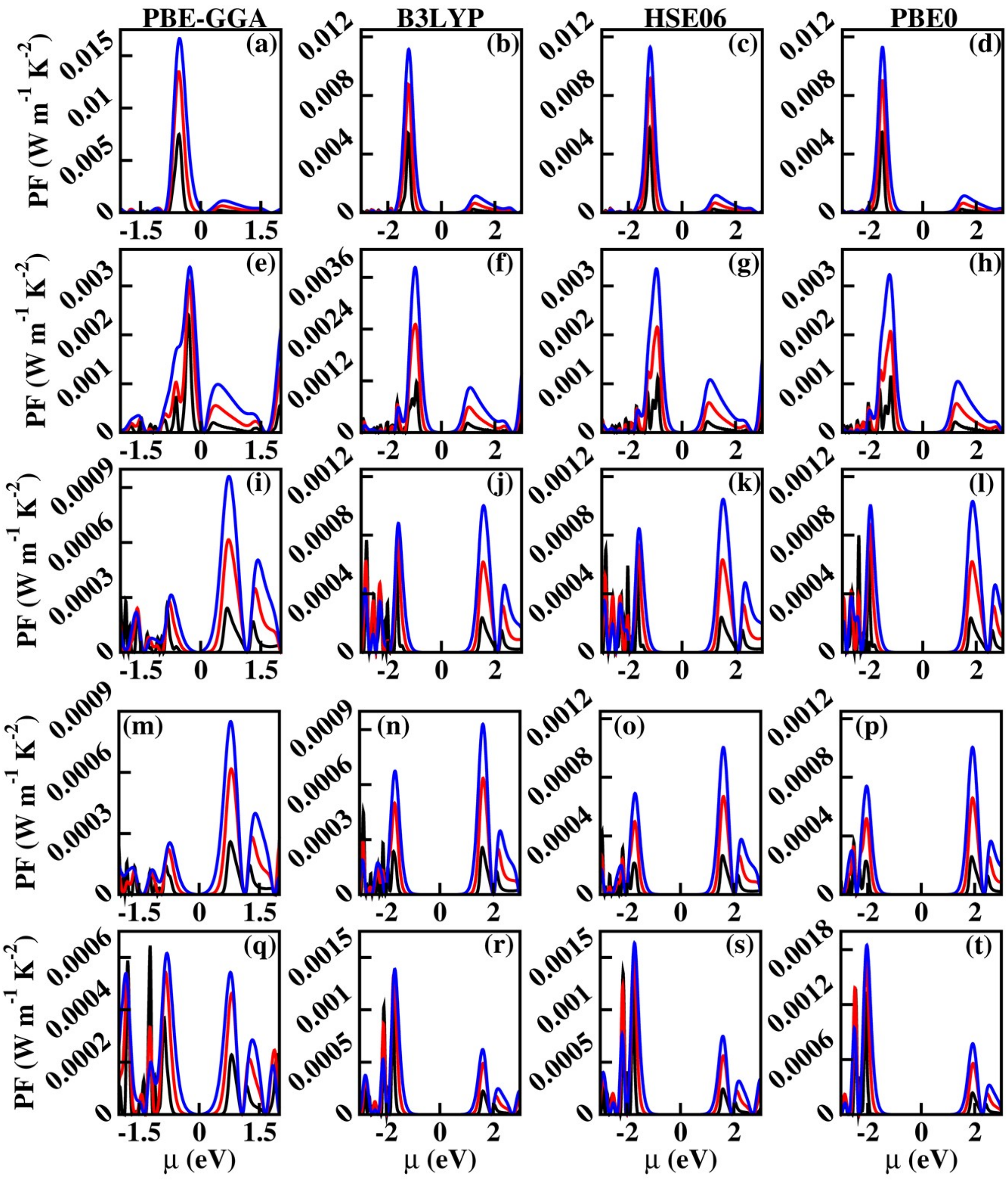

FIG. S12: Calculated power factor (PF) for X_2PbO_3 along x-axis using PBE-GGA, B3LYP, HSE06, and PBE0 functionals: (a-d) X=Li, (e-h) X=Na, (i-l) X=K, (m-p) X=Rb, and (q-t) X=Cs.

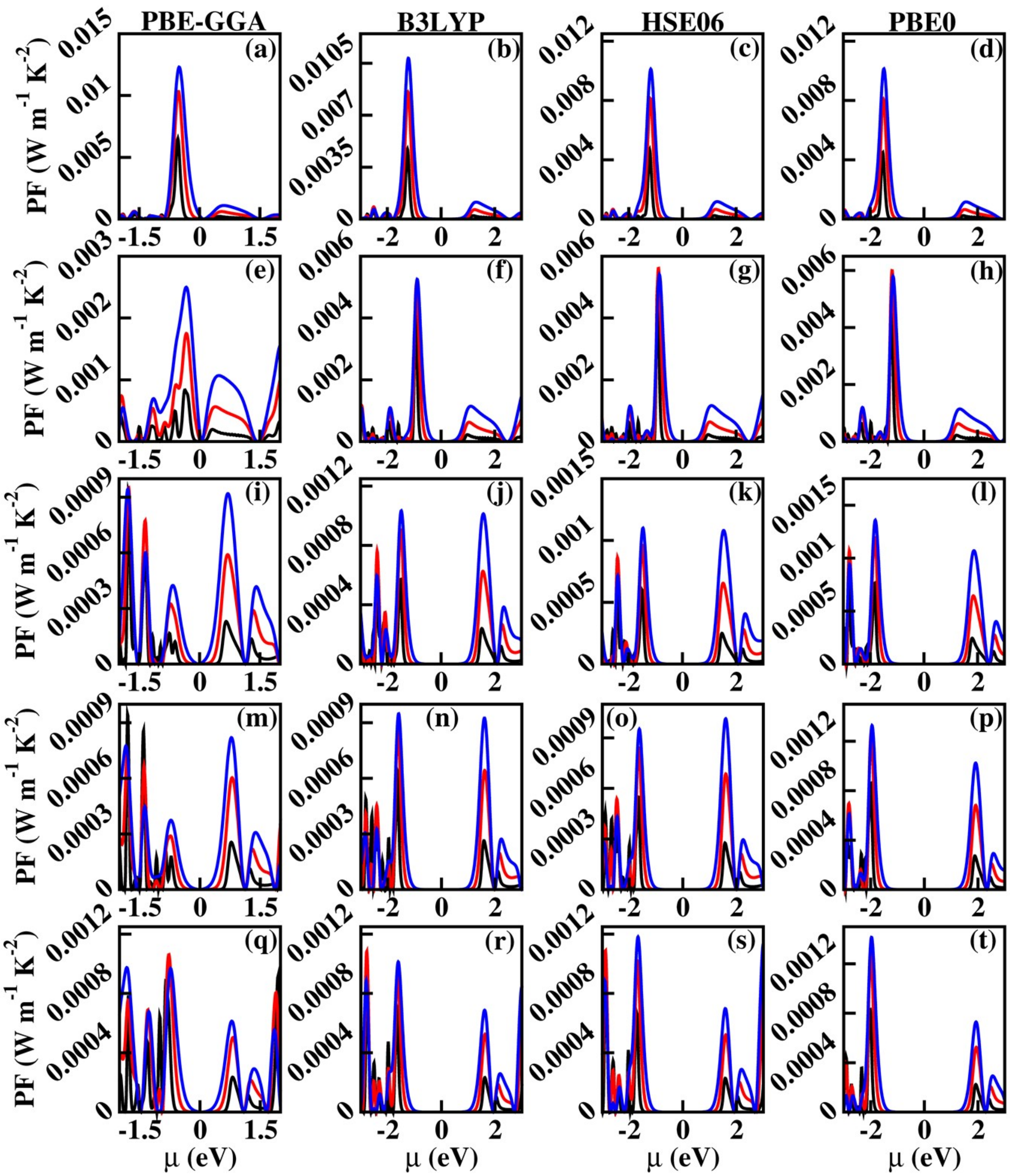

FIG. S13: Calculated power factor (PF) for X_2PbO_3 along y-axis using PBE-GGA, B3LYP, HSE06, and PBE0 functionals: (a-d) X=Li, (e-h) X=Na, (i-l) X=K, (m-p) X=Rb, and (q-t) X=Cs.

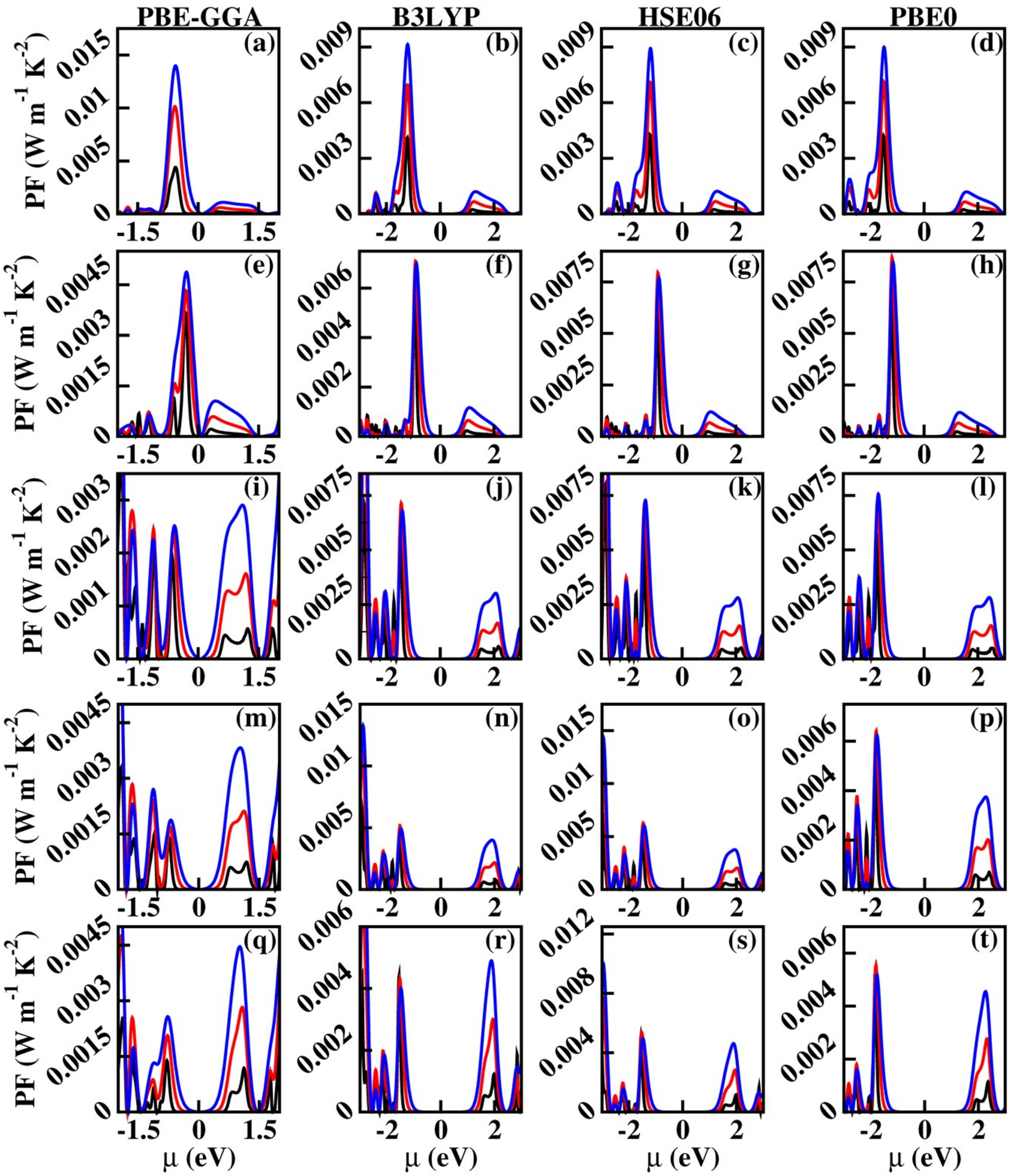

FIG. S14: Calculated power factor (PF) for X_2PbO_3 along z-axis using PBE-GGA, B3LYP, HSE06, and PBE0 functionals: (a-d) X=Li, (e-h) X=Na, (i-l) X=K, (m-p) X=Rb, and (q-t) X=Cs.

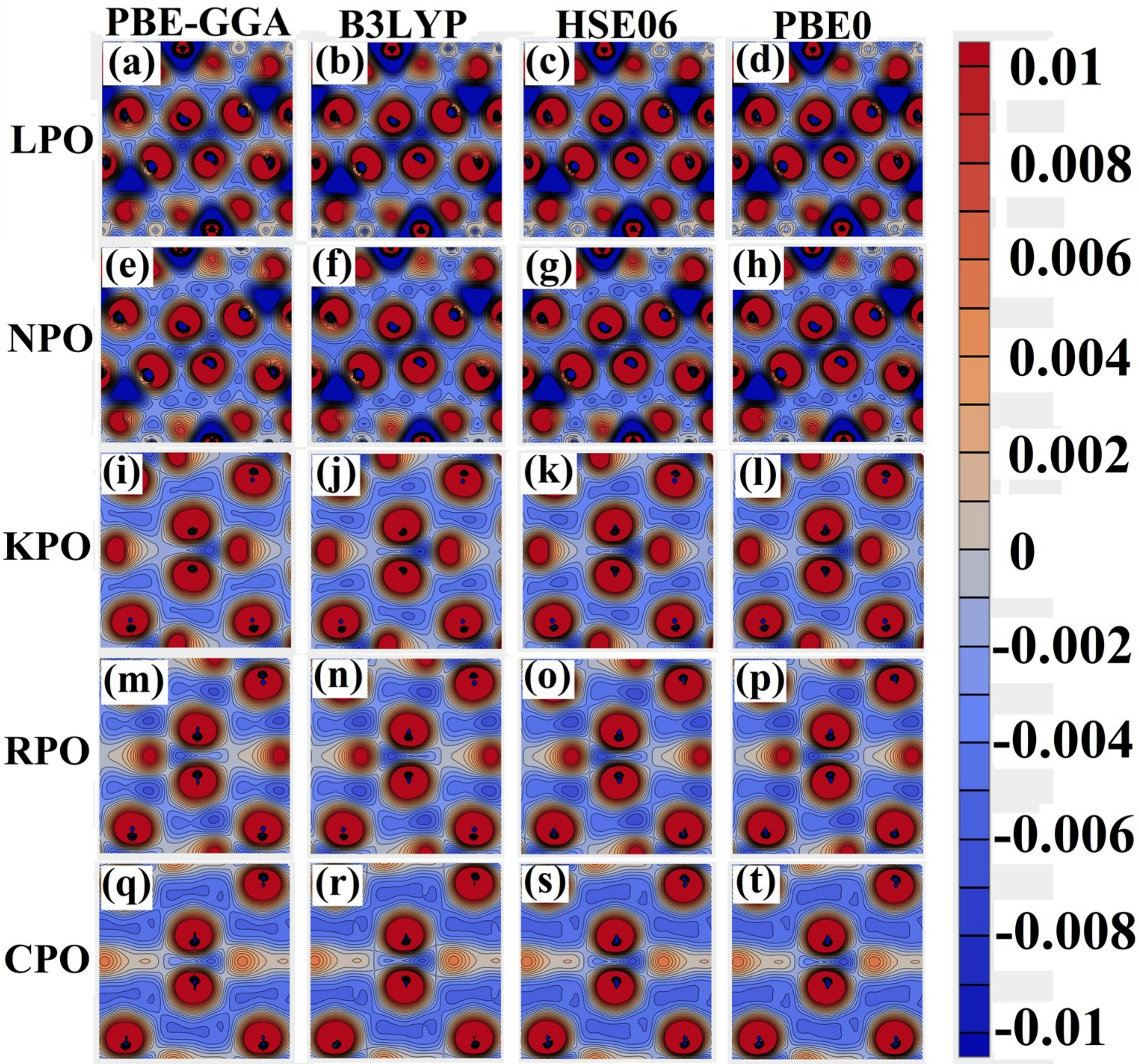

FIG. S15: Calculated difference map of electron charge densities for X_2PbO_3 using PBE-GGA, B3LYP, HSE06, and PBE0 functionals: (a-d) X=Li, (e-h) X=Na, (i-l) X=K, (m-p) X=Rb, and (q-t) X=Cs. Here, a larger red circular region with high density represent O-atom, a smaller red region is Pb-atom and very fainted or empty region located in the interstitial site is X-atom.

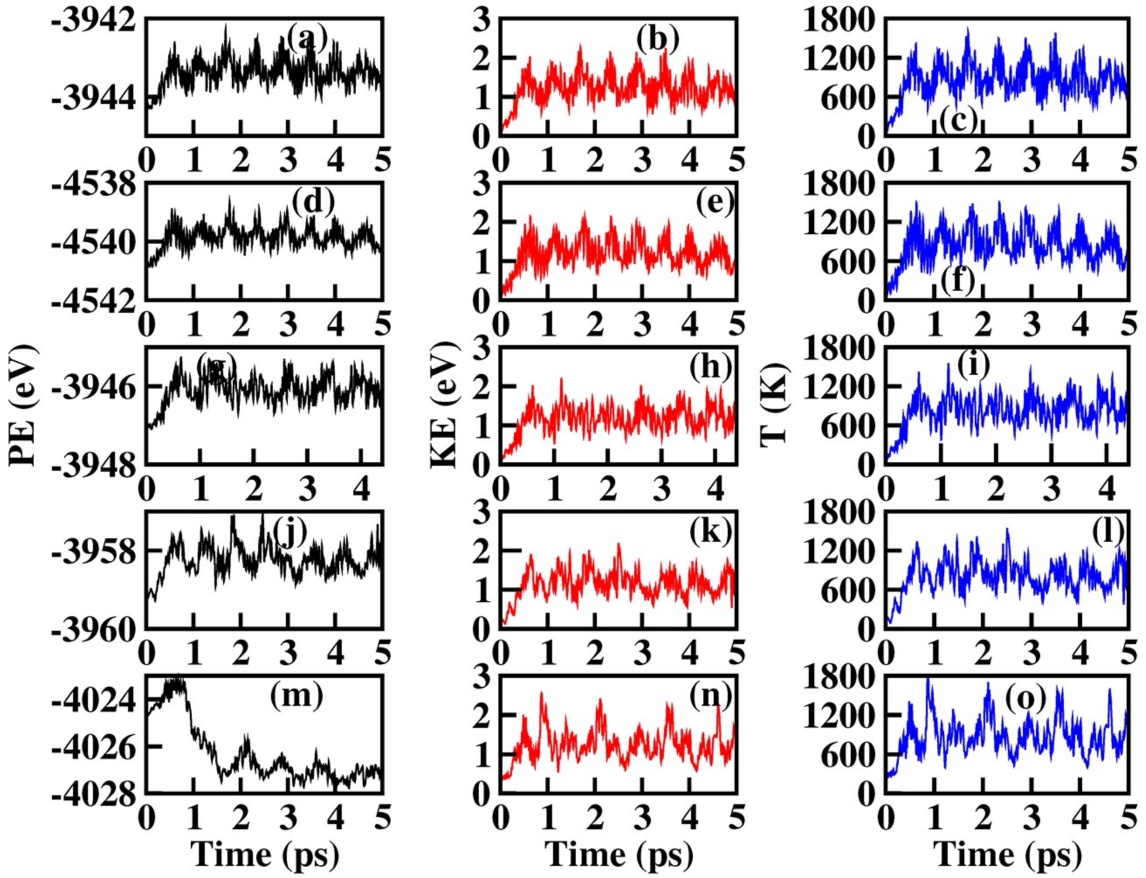

FIG. S16: The potential energy (PE), kinetic energy (KE), and evolution of temperature (T) as a function of time steps calculated using MD-simulation based on nVT-canonical ensemble at T = 850 K: (a-c) X=Li, (d-f) X=Na, (g-i) X=K, (j-l) X=Rb, and (m-o) X=Cs.

Oxygen Vacancy in C*mc*2_1-Li_2PbO_3 and C2/c-X_2PbO_3:

Table S2: Calculated structural parameters for C*mc*2_1-Li_2PbO_3 and C2/c-X_2PbO_3 under oxygen vacancy and electronic band gap.

Compound	a (Å)	b (Å)	c (Å)	α	β	γ	Eg (eV)
Li_2PbO_3	5.65	9.25	5.70	90.00	111.03	90.00	0.86
K_2PbO_3	10.88	7.33	6.14	90.00	90.00	90.00	0.75

In the case of Li_2PbO_3, the creation of an oxygen vacancy leads to a distinct anisotropic lattice response, wherein the lattice constants *a* and *c* exhibit an expansion, while the *b* parameter undergoes a contraction. Conversely, for K_2PbO_3, the introduction of an oxygen vacancy results in a reduction of the *a* lattice constant, accompanied by an increase in both *b* and *c*. These structural distortions can be attributed to the redistribution of local bonding environments and relaxation effects around the vacancy site. In addition to the lattice modifications, the formation of oxygen vacancies induces a noticeable reduction in the electronic band gap (*E*g) of both compounds, reflecting the creation of defect states

and enhanced electronic delocalization. This band gap narrowing suggests that vacancy engineering could significantly influence the electronic and functional properties of X_2PbO_3 systems.

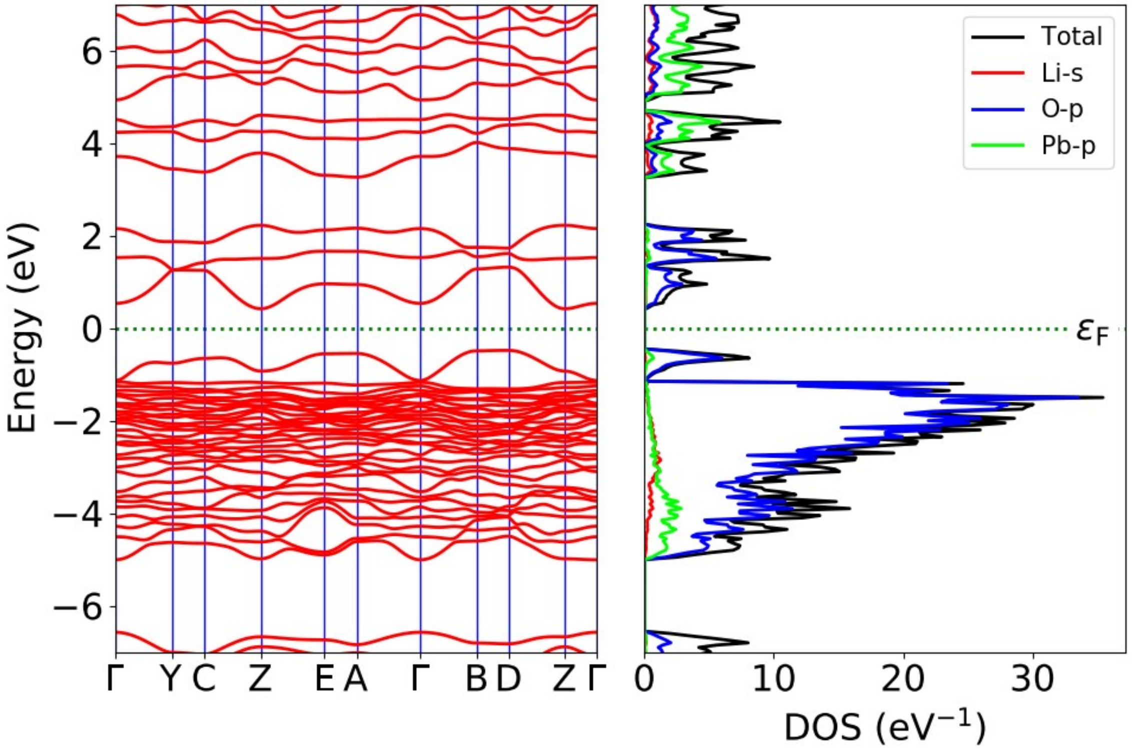

FIG. S17: Band structure and DOS for Li_2PbO_3 with oxygen vacancy calculated using GGA approximation.

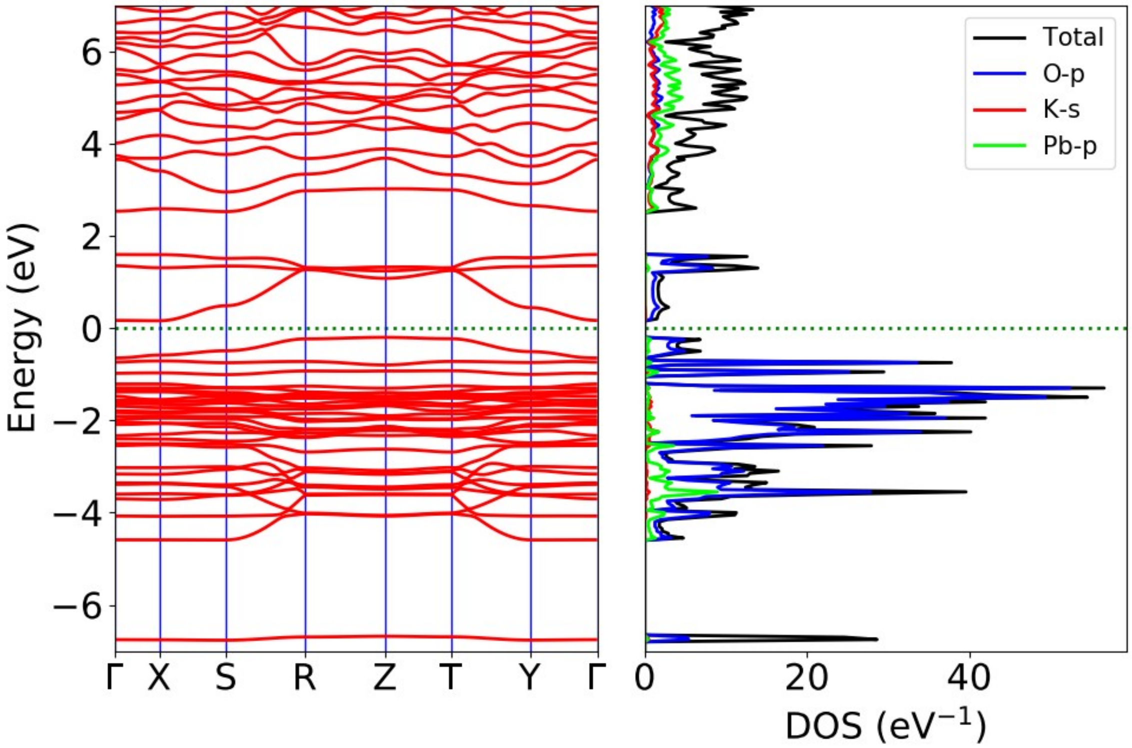

FIG. S18: Band structure and DOS for K_2PbO_3 with oxygen vacancy calculated using GGA approximation.

References:

[1] B. Brazel and R. Hoppe, Über Oxoplumbate(IV): Die Kristallstruktur von TT-Li2PbO3 [1], Zeitschrift fur Naturforschung - Section B Journal of Chemical Sciences 37, 1369 (1982).

[2] R. Hoppe, H.-J. Rhrborn, and H. Walker, Neue Plumbate und Stannate der Alkalimetalle, Die Naturwissenschaften 51, 86 (1964).

[3] R. Hoppe and H. Stöver, Zur Kristallstruktur von Rb2PbO3, Zeitschrift für anorganische und allgemeine Chemie 437, 123 (1977).

[4] P. Panek and R. Hoppe, Zur KistallStruktur von Cs2PbO3, Zeitschrift für anorganische und allgemeine Chemie 393, 13 (1972).
